# Systematic review and quantitative meta-analysis of age-dependent human T-lymphocyte homeostasis

**DOI:** 10.3389/fimmu.2025.1475871

**Published:** 2025-01-27

**Authors:** Victoria Kulesh, Kirill Peskov, Gabriel Helmlinger, Gennady Bocharov

**Affiliations:** ^1^ Research Center of Model-Informed Drug Development, I.M. Sechenov First Moscow State Medical University, Moscow, Russia; ^2^ Marchuk Institute of Numerical Mathematics of the Russian Academy of Sciences (INM RAS), Moscow, Russia; ^3^ Modeling & Simulation Decisions FZ-LLC, Dubai, United Arab Emirates; ^4^ Quantitative Medicines LLC, Boston, MA, United States; ^5^ Institute for Computer Science and Mathematical Modelling, I.M. Sechenov First Moscow State Medical University, Moscow, Russia; ^6^ Moscow Center of Fundamental and Applied Mathematics at INM RAS, Moscow, Russia

**Keywords:** immune aging, T-lymphocytes, systematic review, meta-analysis, healthy subjects

## Abstract

**Objective:**

To evaluate and quantitatively describe age-dependent homeostasis for a broad range of total T-cells and specific T-lymphocyte subpopulations in healthy human subjects.

**Methods:**

A systematic literature review was performed to identify and collect relevant quantitative information on T-lymphocyte counts in human blood and various organs. Both individual subject and grouped (aggregated) data on T-lymphocyte observations in absolute and relative values were digitized and curated; cell phenotypes, gating strategies for flow cytometry analyses, organs from which observations were obtained, subjects’ number and age were also systematically inventoried. Age-dependent homeostasis of each T-lymphocyte subpopulation was evaluated via a weighted average calculation within pre-specified age intervals, using a piece-wise equal-effect meta-analysis methodology.

**Results:**

In total, 124 studies comprising 11722 unique observations from healthy subjects encompassing 20 different T-lymphocyte subpopulations – total CD45+ and CD3+ lymphocytes, as well as specific CD4+ and CD8+ naïve, recent thymic emigrants, activated, effector and various subpopulations of memory T-lymphocytes (total-memory, central-memory, effector-memory, resident-memory) – were systematically collected and included in the final database for a comprehensive analysis. Blood counts of most T-lymphocyte subpopulations demonstrate a decline with age, with a pronounced decrease within the first 10 years of life. Conversely, memory T-lymphocytes display a tendency to increase in older age groups, particularly after ~50 years of age. Notably, an increase in T-lymphocyte numbers is observed in neonates and infants (0 – 1 year of age) towards less differentiated T-lymphocyte subpopulations, while an increase into more differentiated subpopulations emerges later (1 – 5 years of age).

**Conclusion:**

A comprehensive systematic review and meta-analysis of T-lymphocyte age-dependent homeostasis in healthy humans was performed, to evaluate immune T-cell profiles as a function of age and to characterize generalized estimates of T-lymphocyte counts across age groups. Our study introduces a quantitative description of the fundamental parameters characterizing the maintenance and evolution of T-cell subsets with age, based on a comprehensive integration of available organ-specific and systems-level flow cytometry datasets. Overall, it provides the most up-to-date view of physiological T-cell dynamics and its variance and may be used as a consistent reference for gaining further mechanistic understanding of the human immune status in health and disease.

## 1 Introduction

The immune system, which spans across multiple organs, cells, molecules and pathways, is intended to protect the human organism from various pathogens. An essential part of a functional immune system is cell-mediated immunity, which relies on the work of antigen-specific T-lymphocytes. Different populations of T-lymphocytes may act, either directly to eliminate the infectious agent or tumor cell (e.g. cytotoxic CD8+ T-lymphocytes) or indirectly to assist other immune cells in their work (e.g. helper CD4+ T-lymphocytes) (1).

**Figure 1:**
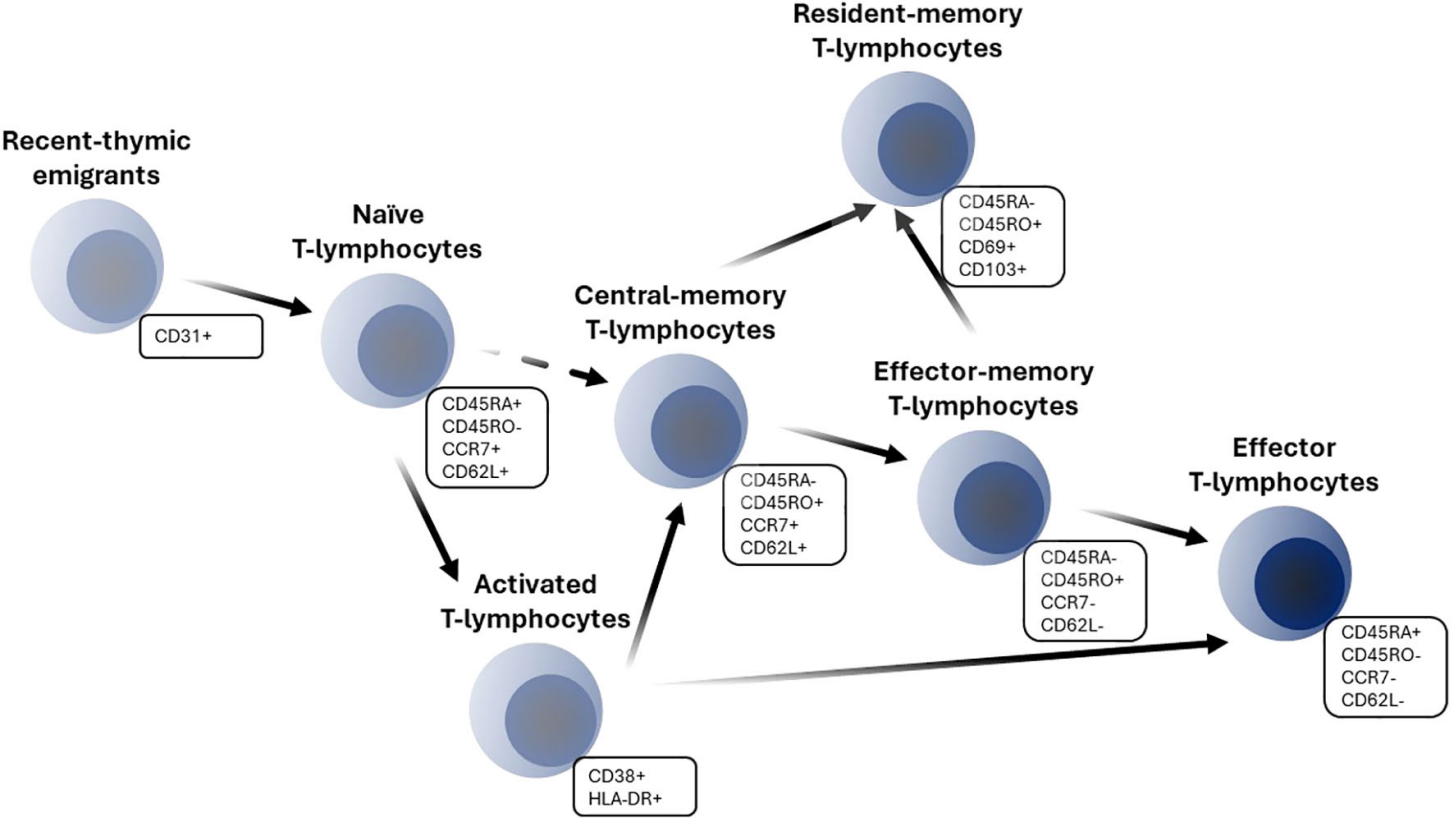
Schematic of T-lymphocyte differentiation.

T-lymphocyte development originates in the thymus and is characterized by an orchestrated staging of maturation processes which lead to T-lymphocyte egress to the peripheral lymphoid system. Peripheral differentiation and expansion of T-lymphocytes include processes such as antigen encountering and self-renewal mechanisms. T-lymphocytes may enter blood circulation as recent-thymic emigrants (RTE), which represent phenotypically and functionally a distinct subpopulation from naïve T-lymphocytes. Human RTE cells can be distinguished from their naïve counterparts by CD31 and PTK7 expressions and reduced immunocompetency (2–4). RTE T-lymphocytes complete their maturation in secondary lymphoid organs and differentiate into mature naïve cells which have not yet encountered an antigen. Naïve T-lymphocytes recirculate continually across blood, lymph and secondary lymphoid organs; they express high levels of the adhesion molecule CD62L and the chemokine receptor CCR7, which regulate lymph homing of cells (5, 6). The encounter of a specific antigen by naïve cells leads to the clonal expansion of activated cells, with a subsequent expression of various cell surface activation and proliferation markers such as CD38, HLA-DR, CD69 and Ki-67 (5). Upon activation, T-lymphocytes acquire the ability to eliminate pathogens or tumor cells and perform their immunological functions. Subsequent processes of T-lymphocyte differentiation to effector and memory cells involve several competing theories; the developmental theory, which favors a progressive differentiation of naïve cells into memory and ultimately effector cells, appears to be more consistent with a host of experimental data (**Figure 1**) (7).

Antigen-specific memory T-lymphocytes make for a persistent pool of cells; they respond more rapidly and robustly than naïve cells upon antigen re-exposure. Such persistence is enabled by long-living memory cells as well as self-renewal mechanisms (8). There are several subpopulations of memory cells, such as central-memory (CM), effector-memory (EM) and resident-memory (RM) T-lymphocytes. CM T-lymphocytes reside mostly in secondary lymphoid organs and express CD62L and CCR7 surface markers, similarly to naïve cells. EM cells represent a circulating subpopulation of T-lymphocytes and can be found in a variety of tissues. They are characterized by the absence of CCR7 and CD62L expression (1, 5). RM cells shape the peripheral immune defense and may arise from either naïve or other memory subpopulations (9). The expression of various markers, such as CD69, CD103, CCR4, α4β1-integrin and others indicates homing of cells in specific organs (10, 11). A separate subpopulation of long-lived stem cell–like memory T-lymphocytes with enhanced self-renewal and multipotency can also be distinguished and characterized via CD95 marker expression (12). Effector helper and cytotoxic T-lymphocytes represent short-lived cells which are directly responsible for carrying out the surveillance functions of an immune response. Effector cells can emigrate to an infection site; they exhibit an opposite phenotype to naïve T-lymphocytes (1). Distinctions among naïve, effector and various memory T-lymphocyte subpopulations are guided not only by CD62L and CCR7 lymph-homing markers, but also by the expression of CD45 isoform variants RO and RA as well as the expression of CD95 (Fas/APO-1), co-stimulatory molecules CD27 and CD28, IL-2Rβ (CD122) and IL-7Rα (CD127) (1, 5). Also, functional discrimination of CD4+ helper and CD8+ cytotoxic T-cells, as well as regulatory T cells have been reported (13–15).

A clear understanding of the relationships between various T-lymphocyte subpopulations as well as the evaluation of their age-dependent homeostasis is necessary, in order to design and optimize novel anti-infectious and T-cell based therapies. The isolation and quantification of various T-lymphocyte subpopulations has become possible due to flow cytometry analyses, following accurate gating strategies and settings (6). The ability to discriminate various subsets of cells based on extracellular and intracellular markers is of importance when assessing dynamic changes in lymphocyte numbers throughout life, as a hallmark of immune aging. Aging processes are at play across all parts of the immune system and comprise several mechanisms, such as thymic involution, shift in hematopoietic stem cells from lymphoid to myeloid potential, increase in the pool of protective memory cells, accumulation of senescent cells, and more (16). Lymphocyte numbers represent markers of immunological health status; thus, absolute counts and percentages of T-lymphocyte subpopulations are measured and compared against reference values in healthy individuals and within specific age categories (17–21). However, these age intervals are usually wide and may not always capture specific differences in immune system maturing and aging processes, particularly in late adulthood. Moreover, for diagnostic purposes, total numbers of lymphocytes and CD4+ or CD8+ T-lymphocytes are routinely measured in blood and not in other organs; also, further specific subpopulations of lymphocytes are not considered. Changes in lymphocyte counts in peripheral blood do not always reflect the full picture of an immune response or a subject’s immunological status, since only ∼2% of total lymphocytes are present in blood (22–24). While there are several approaches to calculate reference values of lymphocyte counts in healthy subjects and to model age-related changes, reference values obtained for specific subpopulations and in different organs present challenges due to limitations in subject numbers enrolled in studies and in study cost (25–31). Gathering the available quantitative information from multiple sources may empower the evaluation of a large repertoire of age-related changes in various T-lymphocyte subpopulations throughout the whole body and make the results more robust.

The primary objective of the present study was to evaluate age-dependent homeostasis of specific T-lymphocyte subpopulations in healthy humans, based on the most comprehensive integration of quantitative data available to date. The corresponding analysis we present here sought to characterize age-related changes in various T-lymphocyte subpopulations, from neonates to centenarians, and to obtain reference values (in absolute terms and percentages) in blood and other organs within narrowest age intervals. Our meta-analysis of T-lymphocyte age-dependent homeostasis addresses issues relevant to the quantitative description of immune aging in various organs; it captures differences in numbers for a wide range of T-lymphocyte types under homeostasis.

The paper is organized as follows: Section 2 describes the data collection strategy, the workflow of data handling, and detailed aspects of our meta-analysis methodology. Section 3 presents the integrative database, overall, along with an exploration of age-dependent healthy human condition homeostasis and our results of weighted average calculations. Sections 4 to 5 provide, respectively, a discussion and conclusions.

## 2 Materials and methods

### 2.1 Study eligibility and data collection

A systematic literature search was performed to identify all relevant data sources featuring clinical data on T-lymphocyte numbers. The PubMed database and Google Scholar were explored using the following keywords: “CD4”, “CD62L”, “CD45RO”, “CD45RA”, “naïve”, “central-memory”, “effector-memory”, “effector”, “human”, “healthy”, “flow cytometry”. Additional PubMed searches as well as references from the initially identified research papers and review articles were investigated further, to uncover all relevant quantitative information, particularly on specific T-lymphocyte subpopulations in lymph nodes. The exact queries used can be found in **Supplementary Table 1**. All identified records were assessed on the basis of publication title and abstracts focused on the presence of human data and control arm with healthy subjects in the study. Only publicly available text in English language was inspected. If an abstract was considered valid for inclusion into the analysis, the original publication was investigated to inspect observations on T-lymphocyte subpopulations of interest and subjects’ age records. All selected data sources were assessed for potential cross-publication overlap, prior to digitizing. Results from the selected data sources were digitized and integrated into the database. The latest database update was performed on July 11^th^, 2024.

### 2.2 Outcome measures

The following features were digitized and added to a Microsoft Excel database: population or subject characteristics such as age and health condition; T-lymphocyte measurement characteristics such as reported cell phenotypes, gating strategy used for flow cytometry analysis, units and direct measurements; and central tendency and variability measures approaches used for grouped data. Observations from subjects with a health condition that would potentially affect the immune system (i.e., the presence of Cytomegalovirus (CMV), Epstein–Barr virus or other infections) were not digitized or were excluded, at the exploratory analysis stage. Twenty (20) distinct T-lymphocyte subpopulations were built into the database, according to the reported cell phenotypes and gating strategies. The outcomes consist of total and specific T-lymphocyte subpopulations. Total subpopulations included total lymphocytes and CD3+ lymphocytes (T-lymphocytes), while specific subpopulations were represented by CD4+ and CD8+ T-lymphocytes: recent-thymic emigrants, naïve, activated, total-memory, central-memory, effector-memory and resident-memory cells. A schematic of T-lymphocyte subpopulation transitions is presented in **Figure 1**. The selected gating strategy for each T-lymphocyte subpopulation is presented in **Supplementary Table 2**. Aside from the exact numbers of T-lymphocyte subpopulations, we also digitized data on published CD4+/CD8+ ratios – an important indicator of immune system function.

Overall, we extracted individual and grouped observations on 21 parameters of immune homeostasis (20 T-lymphocyte subpopulations and CD4+/CD8+ ratio), which are presented in absolute and relative values.

### 2.3 Statistical analyses

Weighted averages as generalized estimates were calculated for each outcome using an equal-effect meta-analysis approach for pre-specified age intervals:


(1) $${\text{y}}_{\text{i}}=\;\text{θ}+{\text{ϵ}}_{\text{i}}$$

where i=1,…,k denotes independent studies for which outcomes were available for the age interval, $y_{i}$ denotes the observed outcome measure in the $i^{th}$ study, θ is the true outcome of the k studies, and $\epsilon_{\mathrm{i \sim}}N\left(0,\;\sigma^{2}\right)$ represents the sampling error (32).

Weighing was determined according to subject numbers. The conventional inverse variance weighing method was not implemented, since the assumption of a proportional decrease in variance with an increase in subject number was not met. A comparison of two weighing methods was performed by visual inspection of scatter plots:


(2) $$\hat{\text{θ}}=\frac{\sum_{\text{i}=1}^{\text{k}}{\text{ω}}_{\text{i}}{\text{y}}_{\text{i}}}{\sum_{\text{i}=1}^{\text{k}}{\text{ω}}_{\text{i}}}$$

where $\hat{\theta }$ is the weighted average of the true outcomes in the k studies; the weight was computed as $\omega_{i}=\frac{n_{i}}{\sum_{i=1}^{k}n_{i}}$ , where n is the number of subjects in the $i^{th}$ study.

To implement a standard meta-analysis for continuous outcomes and meet the requirement of information on mean and standard deviation (SD), the data were transformed to obtain this information. Missing mean and SD were imputed based on the available central tendency and variability information, according to the Wan et al. transformation equation, with the assumption of normally distributed outcomes (Equations 3, 4) (33, 34). Discrepancies between raw and transformed grouped data were evaluated.


(3) $$\bar{\text{y}}\approx \frac{{\text{q}}_{1}+\text{m}+{\text{q}}_{3}}{3}$$

(4) $${\text{s}}_{\text{x}}\approx \frac{{\text{q}}_{3}-{\text{q}}_{1}}{2\Phi^{-1}\left(\frac{0.75\text{n}-0.125}{\text{n}+0.25}\right)}$$

where $\bar{y}$ is the estimate of the missing mean value for the outcome, $s_{x}$ is the estimate of the missing SD value, $q_{1}$ and $q_{3}$ represent the ( α ) and ( 100−α ) percentiles, respectively ( α = 2.5%, 10%, 25% (interquartile range)), m is the median, n is the sample size, and ${\text{Φ}}^{-1}\left(z\right)$ is the upper $z^{th}$ percentile of the standard normal distribution.

Measurements of cell counts and concentrations were standardized to obtain units in “cells/µL”. Mass (cells/g) and area density (cells/mm^2^) numbers were also standardized, unit-wise, yet these were not included in the final analysis due to smaller numbers of observations. In addition, observations were grouped based on the organ from which the measurements were obtained.

The presence of both individual and grouped data in the database poses the challenge of combining these data for weighted average calculations. Mean and SD values were computed for individual subject observations in the pre-specified age intervals: from 0 to 6 months with a 3-month step; from 6 months to 1.0 year; from 1.0 to 2.5 years; from 2.5 to 5.0 years; and from 5.0 to 115 years with a 5-year step. Computed estimates based on such individual subject-level data were then grouped for further analysis.

Data binning for weighted average calculations was guided based on both physiological and statistical considerations. The selected strategies for data binning are presented in **Supplementary Table 3**. Age intervals were selected based on the life cycle grouping, with more frequent divisions for the first years of life (2 to 5 intervals were considered within the 0-to-5 year age group). The selection of age intervals was also guided by a requirement of at least two (2) observations falling into a given interval.

Heterogeneity was evaluated via visual inspection of funnel plots as well as an estimation of publication bias (Egger’s test) for each outcome and each age interval.

### 2.4 Software

Data digitization was performed using *WebPlotDigitizer* version 4.7 (for scatter and bar plots) and *jcpicker* version 6 (for heat maps). Data processing, visualization (main packages: *tidyverse*, *cowplot*, *ggpubr*, *ggrepel*) and meta-analyses (package: *metafor*) were performed in the *R Statistics* software, version 4.2.3 (*R-project*, www.r-project.org).

## 3 Results

### 3.1 Study selection and database overview

**Figure 2:**
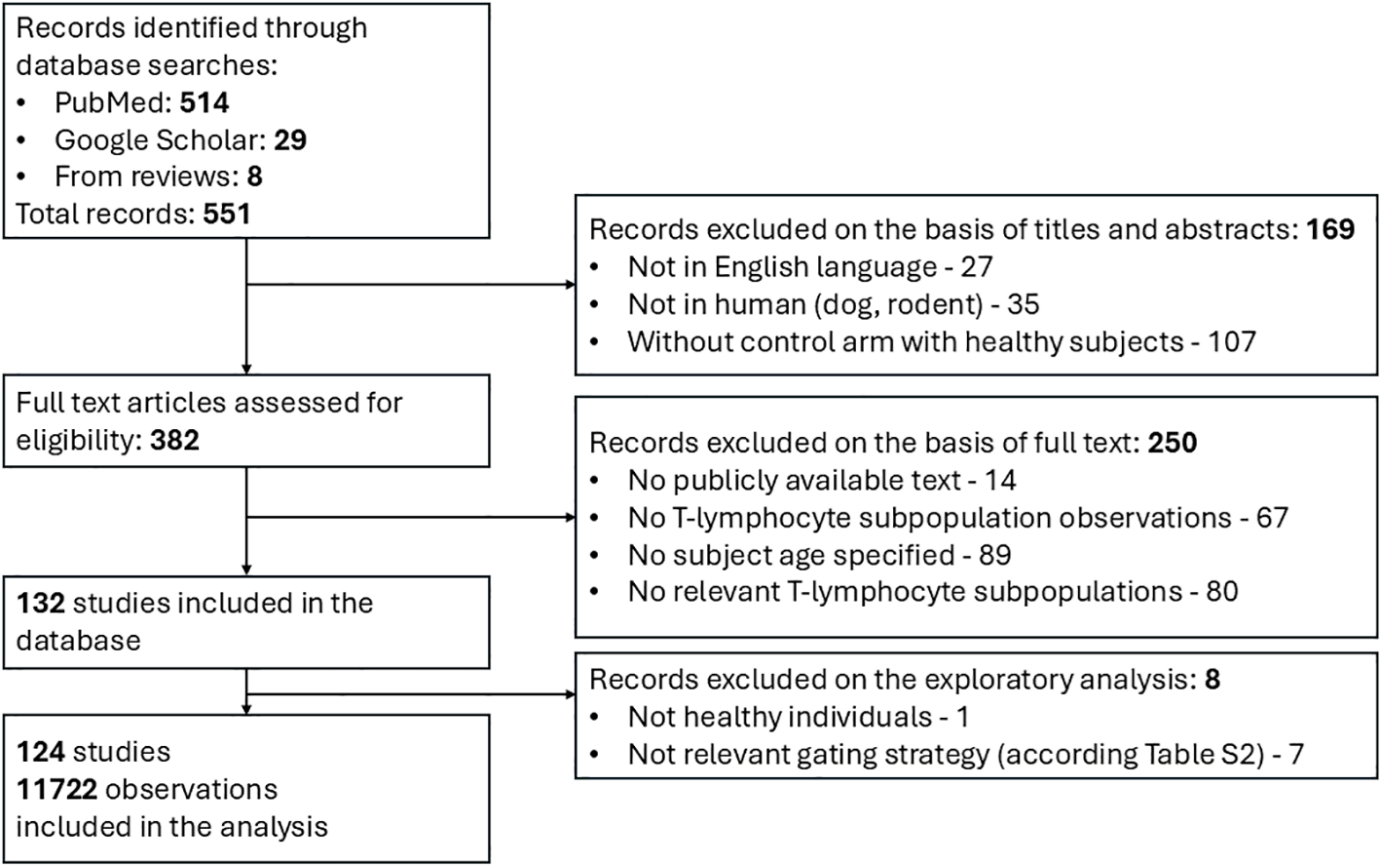
Flow diagram for search and selection of clinical studies featuring quantitative data on T-lymphocyte homeostasis.

The systematic search in PubMed and Google Scholar resulted in 551 potentially relevant records of quantitative data on T-lymphocyte age-dependent homeostasis. A majority of records (514) were identified via the PubMed database search, while 29 and 8 records were extracted, respectively, from the Google Scholar search and review articles. 169 studies were discarded based on the screening of titles and abstracts for the following reasons: not available in English language; irrelevant species (dog, rodent; not human); and absence of control arms with healthy subjects. In total, 382 articles proceeded to the full-text analysis stage; this resulted in the exclusion of 250 records due to: unavailability of text access in the public domain; absence of quantitative data on T-lymphocyte subpopulations; irrelevance of the subpopulation in the present analysis and/or no specification according to subject age. As a result, 132 clinical studies fully satisfied the inclusion criteria and were incorporated into the final database. The subsequent exploratory analysis resulted in the exclusion of an additional 8 studies which had not been conducted in fully healthy individuals or according to relevant gating strategies (see Section 2.2 and **Supplementary Table 2**). The flowchart leading to article and study selection is presented in **Figure 2**.

The final dataset for the analysis comprised a total of 124 studies with 11,722 unique observations on T-lymphocyte numbers (10, 18, 25, 30, 31, 35–153). Both individual and grouped data are presented in the dataset, with the number of subjects ranging from 3 to 263. All observations were stratified based on seven (7) physiological organs: blood, lymphoid organs (consisting of spleen, Peyer patches, bone marrow, lung, inguinal, mesenteric, iliac, pelvic and cervical lymph nodes), lung (consisting of lung tissue, lung lamina propria and broncho-alveolar lavage fluid), gastro-intestinal tract (consisting of jejunum, ileum, colon, gut lamina propria and rectum), thymus, kidney, liver and “other tissues” (consisting of joint gut and lung lamina propria, cerebrospinal fluid and colostrum).

The percentage distribution of individual and grouped data in absolute and relative terms, for each of the 21 outcomes, is shown in **Supplementary Table 2**. Individual data in absolute values of cell counts constituted the majority of the total lymphocyte subpopulation data (94.0% for total lymphocytes and 72.6% for CD3+ T-lymphocytes) and several specific T-lymphocyte subpopulations (66.3% and 65.5% for CD4+ and CD8+ total T-lymphocytes, 50.2% and 57.9% for CD4+ and CD8+ memory T-lymphocytes). Other specific subpopulation data consisted predominantly of individual observations in relative values. Data for CD8+ RTE cells were found only in relative values, most likely because of inherent difficulties in distinguishing clear CD8+ RTE differentiation within the common phenotypic CD31 marker (2). In contrast, observations in absolute values of CD4+ cells accounted for more than 15% of the total CD4+ RTE observations. Data for activated CD38+ HLADR+ T-lymphocytes are presented mostly for subject groups, not for individuals, for both CD4+ and CD8+ populations (with exception of two (2) observations in relative values for CD8+ subpopulation). Observations of resident-memory lymphocytes were available in relative values only.

**Figure 3:**
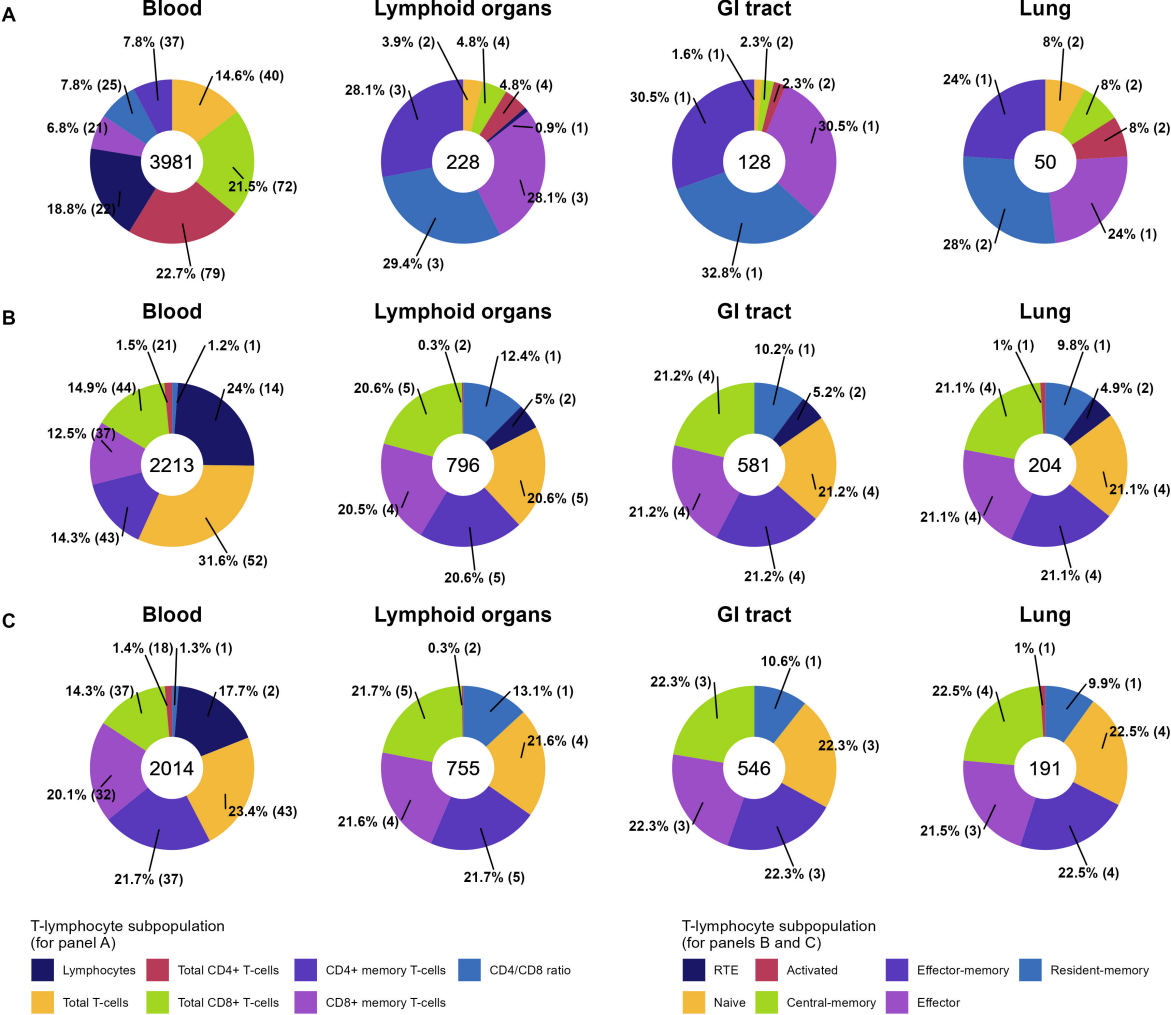
Percentage distributions of unique observations of total **(A)**, specific CD4+ **(B)**, and specific CD8+ **(C)** T-lymphocyte subpopulations in the final dataset across 4 physiological organs (35 observations from “Kidney”, “Liver”, “Thymus”, “Other tissues” are not included here but can be found in **Supplementary Figure 1**). Numbers within donut charts represent the total numbers of observations. The proportion of each T-lymphocyte subpopulation in an organ is provided as a percentage, with the number of unique studies given within the brackets.

Distributions of T-lymphocyte subpopulation observations in the final dataset are shown in **Figure 3**. The majority of the quantitative data for both total and specific subpopulations are presented for blood (3981, 2213 and 2014 observations for total, specific CD4+, CD8+ subpopulations, respectively), the most common and convenient measurement compartment. Blood was enriched in total CD3+ (14.6%), CD4+ (22.7%) and CD8+ (21.5%) total T-lymphocyte observations, while these subpopulations were sparser, as compared to memory counterparts, in other organs. CD4+/CD8+ ratio data are presented for all considered physiological organs. The most represented specific T-lymphocyte subpopulations were naïve, central-memory, effector-memory and effector T-lymphocytes, comprising >20%, >14%, >14% and >12% of observations in the dataset, respectively, for each organ. The least represented subpopulations for each organ were activated (1.5% and 1.0% for CD4+ and 1.4% and 1.0% for CD8+ of observations in blood and lung, respectively) and resident-memory T-lymphocytes, which were present mostly at peripheral sites (CD4+ RM: 1.2% in blood vs. 12.4%, 10.2% and 9.8% in lymphoid organs, GI tract and lung, respectively; CD8+ RM: 1.3% in blood vs. 13.1%, 10.6% and 9.9% in lymphoid organs, GI tract and lung, respectively).

### 3.2 Age-dependent homeostasis of T-lymphocytes in organs and tissues

**Figure 4:**
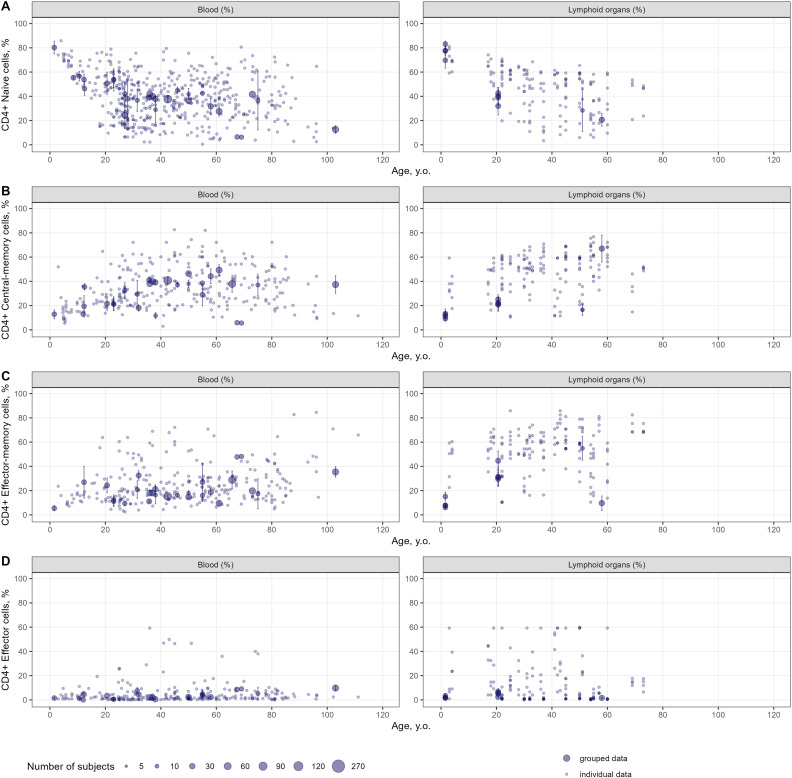
Age-dependent homeostasis of CD4+ T-lymphocyte subpopulations in blood and lymphoid organs (values shown are relative to total CD4+ T-lymphocytes) [**(A)** – naïve (studies: 36; observations: 509); **(B)** – central-memory (studies: 33; observations: 290); **(C)** – effector-memory (studies: 32; observations: 277); **(D)** – effector (studies: 26; observations: 236)]. Dots represent individual data, dots with error bars represent the means with 95% CIs of the grouped data, dot diameters indicate subject numbers per group.

**Figure 5:**
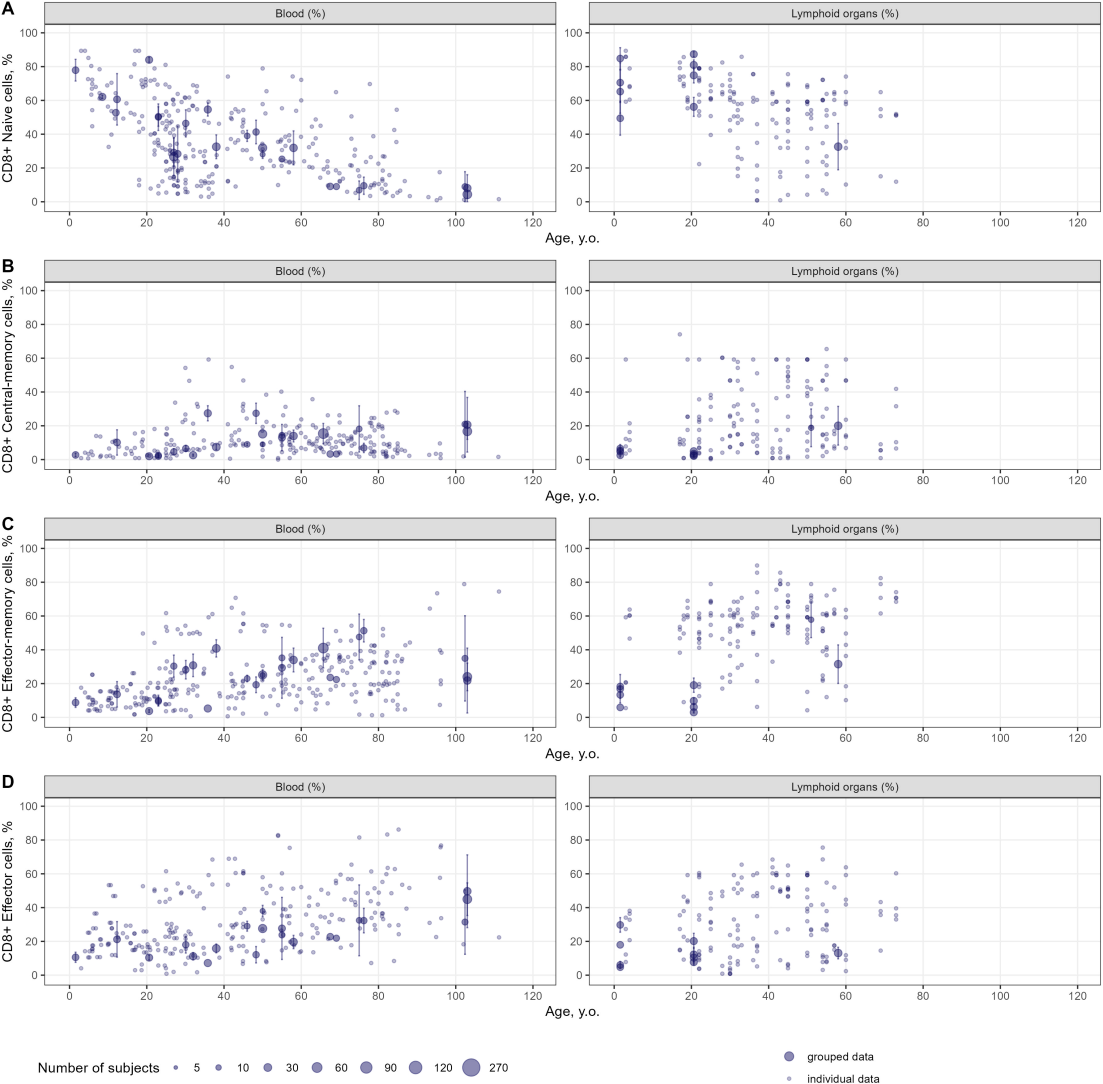
Age-dependent homeostasis of CD8+ T-lymphocyte subpopulations in blood and lymphoid organs (values shown are relative to total CD8+ T-lymphocytes) [**(A)** – naïve (studies: 28; observations: 312); **(B)** – central-memory (studies: 27; observations: 255); **(C)** – effector-memory (studies: 27; observations: 404); **(D)** – effector (studies: 24; observations: 371)]. Dots represent individual data, dots with error bars represent means with 95% CIs of the grouped data, dot diameters indicate subject numbers per group.

Age-dependent homeostasis of T-lymphocyte subpopulations was explored to evaluate the kinetics of age-related changes in cell counts and percentages as well as the heterogeneity in the extracted data. Age-related changes in percentages of specific subpopulations of CD4+ and CD8+ T-lymphocytes in the blood and lymphoid organs are shown in **Figures 4** and **5**, respectively. Individual data on lymphocyte percentages were consistent with grouped data, however, variability increased with age in almost all subpopulations. Percentages of naïve CD4+ and CD8+ T-lymphocytes decreased with age, while percentages of central-memory, effector-memory, and effector cells increased – a result consistent with the age-related shift in cell phenotypes to more differentiated ones (16). No differences in age-dependent homeostasis of specific subpopulations were observed between blood and lymphoid organs. Age-related changes in cell counts for total and specific subpopulations of T-lymphocytes are presented in **Supplementary Figures 2**-**4**.

CD4+/CD8+ ratios tended to increase with age in blood, lymphoid organs, lung and GI tract (see **Supplementary Figure 5**) – an indication of cytotoxic CD8+ T-cells depleting more rapidly with age, as compared to CD4+ helper T-cells. In each organ considered, most CD4+/CD8+ ratios fell within the range of 1.0 and 5.0.

Age-related changes in CD4+ and CD8+ resident-memory T-lymphocytes were explored, as shown in **Supplementary Figures 6** and **7**, respectively. Only one data source with observations on CD45RO+CD69+ and CD45RO+CD103+ cell percentages was selected into the database, based on the gating strategy used (10). CD69 represents an early activation marker and marker of tissue residency, whose expression is upregulated by memory T-cells in all tissues, while the CD103 integrin is associated with mucosal and barrier tissue sites and is more specifically attributed to CD8+ resident-memory cells (154). Blood percentage counts of both CD4+ and CD8+ resident-memory cells were close to zero (less than 10% for the majority of observations), indicative of tissue residency. In contrast, CD4+ and CD8+ CD69+ resident-memory cells constituted a large portion of memory cells in lymphoid organs (average for all ages: ~64.8% for CD4+ and ~68.9% for CD8+), lung (average for all ages: ~66.9% for CD4+ and ~69.0% for CD8+) and GI tract (average for all ages: ~78.3% for CD4+ and ~80.7% for CD8+). CD103+ expression was notably higher on CD8+ vs. CD4+ CD45RO+ memory T-lymphocytes (lymphoid organs: 15.8% vs. 0.7%; lung: 23.0% vs. 2.9%; GI tract: 68.9% vs. 18.3%).

### 3.3 Meta-analysis of T-lymphocyte age-dependent homeostasis

**Figure 6:**
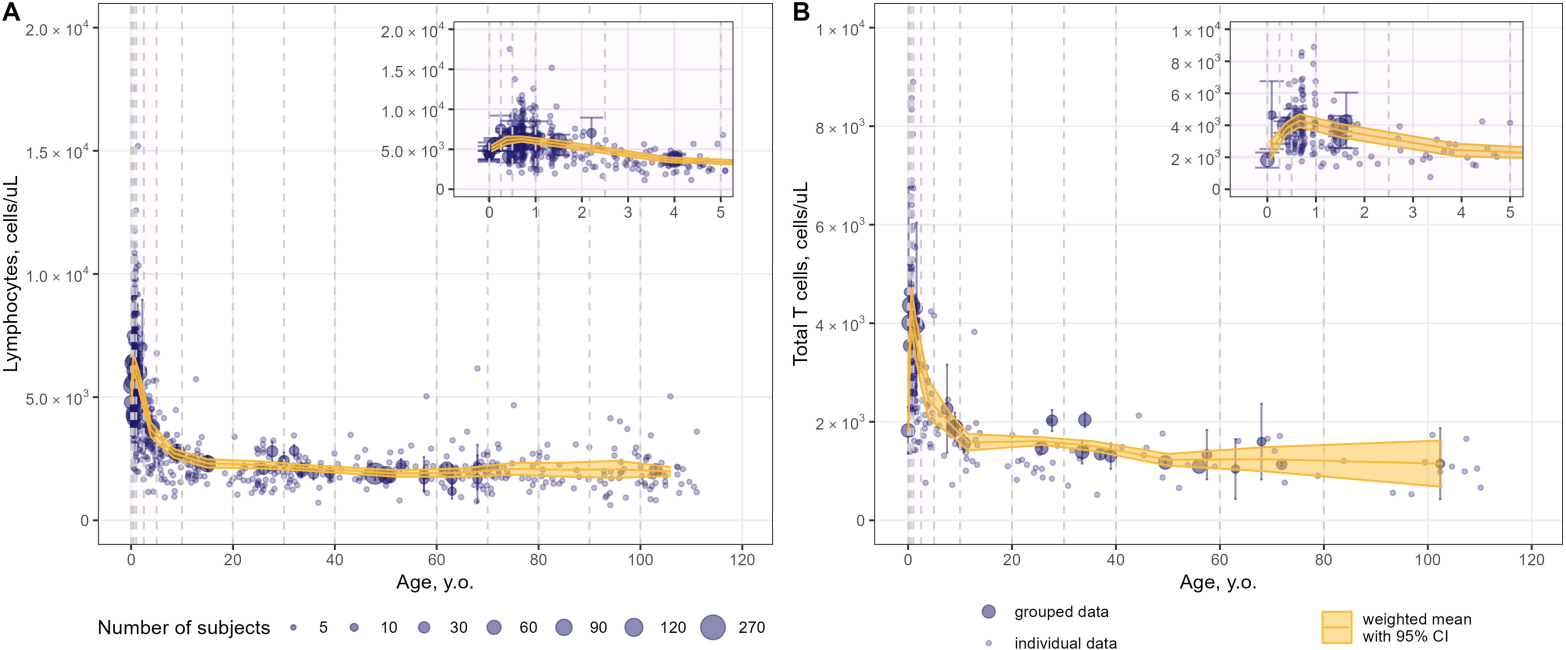
Meta-analysis of age-dependent homeostasis of total lymphocyte subpopulations in blood [**(A)** – total lymphocytes (studies: 21; observations: 749); **(B)** – total CD3+ T-lymphocytes (studies: 23; observations: 482)]. Numbers represent absolute values. Dots represent individual data, dots with error bars represent means with 95% CIs of the grouped data; yellow solid lines with shaded area represent weighted means with 95% CIs for each age bin (delineated by vertical dashed lines); dot diameters indicate subject numbers per group; purple shaded areas represent data for neonates, infants and toddlers (0 to 5 years of age).

**Figure 7:**
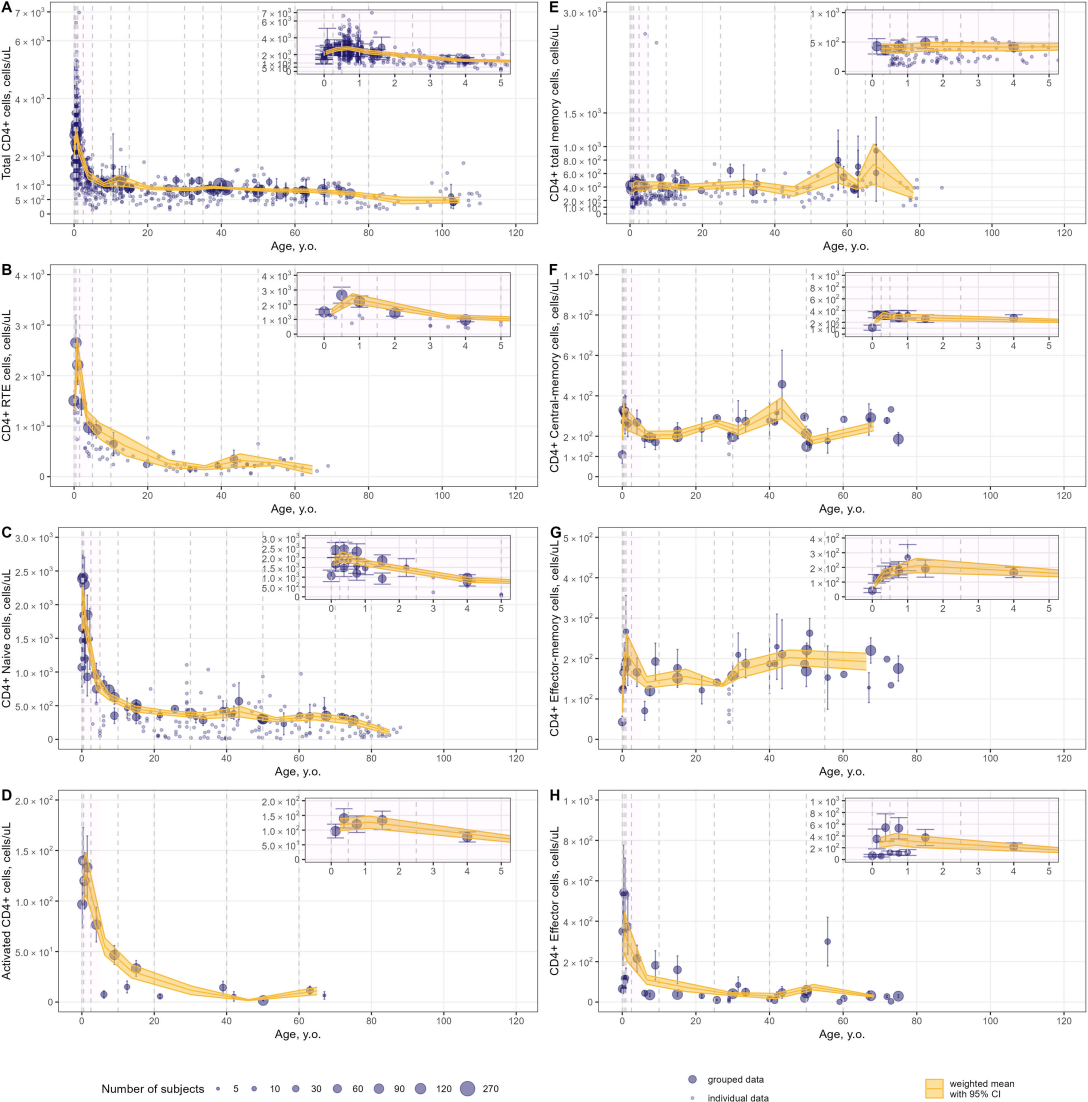
Meta-analysis of age-dependent homeostasis of CD4+ T-lymphocyte subpopulations in blood [**(A)** – total CD4+ (studies: 46; observations: 695); **(B)** – CD4+ RTE (studies: 8; observations: 93); **(C)** – CD4+ naïve (studies: 19; observations: 190); **(D)** – CD4+ activated (studies: 5; observations: 15); **(E)** – CD4+ total memory (studies: 15; observations: 240); **(F)** – CD4+ central-memory (studies: 13; observations: 40); **(G)** – CD4+ effector-memory (studies: 13; observations: 40); **(H)** – CD4+ effector T-lymphocytes(studies: 13; observations: 40)]. Numbers represent absolute values. Dots represent individual data, dots with error bars represent means with 95% CIs of the grouped data; yellow solid lines with shaded area represent weighted means with 95% CIs for each age bin (delineated by vertical dashed lines); dot diameters indicate subject numbers per group; purple shaded areas represent data for neonates, infants and toddlers (0 to 5 years of age).

**Figure 8:**
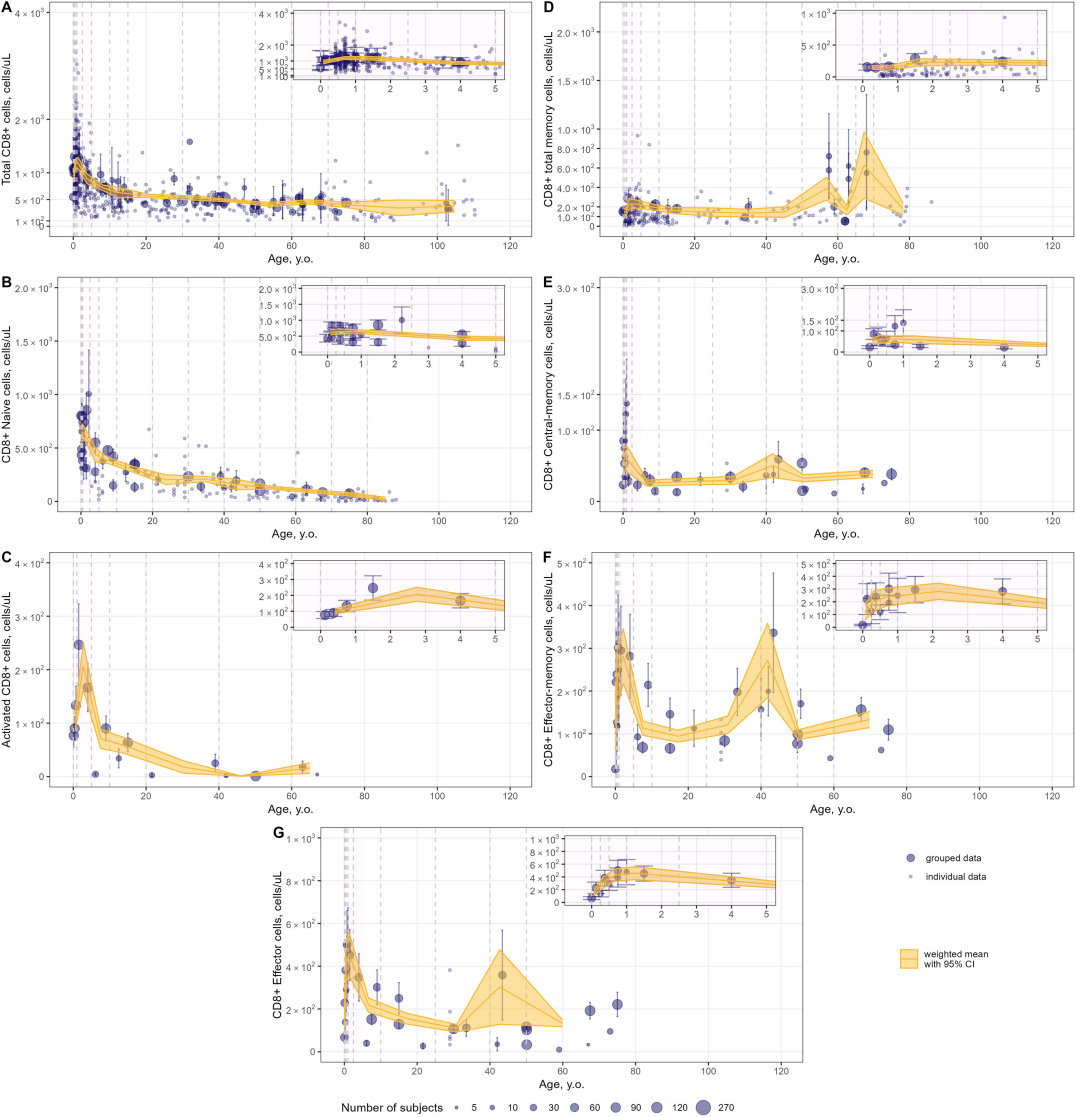
Meta-analysis of age-dependent homeostasis of CD8+ T-lymphocyte subpopulations in blood. [**(A)** – total CD8+ (studies: 40; observations: 647); **(B)** – CD8+ naïve (studies: 16; observations: 160); **(C)** – CD8+ activated (studies: 5; observations: 15); **(D)** – CD8+ total memory (studies: 10; observations: 245); **(E)** – CD8+ central-memory (studies: 11; observations: 34); **(F)** – CD8+ effector-memory (studies: 11; observations: 34); **(G)** – CD8+ effector T-lymphocytes(studies: 9; observations: 33)]. Numbers represent absolute values. Dots represent individual data, dots with error bars represent means with 95% CIs of grouped data; yellow solid lines with shaded area represent weighted means with 95% CIs for each age bin (delineated by vertical dashed lines); dot diameters indicate subject numbers per group; purple shaded areas represent data for neonates, infants and toddlers (0 to 5 years of age).

Weighted averages of counts were calculated for each T-lymphocyte subpopulation in the pre-specified age intervals. The meta-analysis results for total lymphocyte and CD3+ T-lymphocyte age-related count changes in blood are shown in **Figure 6**; for specific CD4+ and CD8+ subpopulations, age-dependent homeostasis is presented in **Figures 7** and **8**, respectively.

Blood counts of most T-lymphocyte subpopulations declined with age. A substantial decrease in T-lymphocyte counts was observed in the first 10 years of life, followed by a slower decay in subsequent years. Memory T-lymphocyte subsets (total, central-memory, effector-memory CD4+ and CD8+) tended to increase at older ages after approximately 50 years of age, consistent with the concept of immune aging.

Intriguingly, an increase in T-lymphocyte counts was found, upon close examination of age profiles, in nearly all subpopulations in neonates, infants and toddlers (0 to 5 year-old age groups; see a detailed representation in **Figures 6**–**8** inserts). A maximal count in total subpopulations was reached in the 6-to-12 month of age group, with 6280 cells/µL (95% CI: 5950–6611) of total lymphocytes and 4291 cells/µL (95% CI: 3889–4693) of total CD3+ T-lymphocytes. However, an increase in blood count in neonates and infants (0–6 months) was less discernible for total memory CD4+ (**Figure 7E**) and CD8+ (**Figure 8D**) T-lymphocytes vs. total CD4+ (**Figure 7A**) and CD8+ (**Figure 8A**) T-lymphocytes. Moreover, maximal blood counts of memory subpopulations at younger ages were reached later vs. total counterparts (1.0–2.5 years of age for memory CD4+ and CD8+ cells vs. 6.0–12 months of age for total CD4+ and CD8+ cells).

When considering specific subpopulations of memory T-lymphocytes, differences in times-to-maximal counts during childhood were observed. Maximal blood counts of effector-memory T-lymphocytes were achieved at a later age (CD4+: 1.0–2.5 years of age; CD8+: 1.0–5.0 years of age) vs. central-memory T-lymphocytes (CD4+: 3.0–6.0 months of age; CD8+: 6.0–12 months of age). Also, differences in maximal counts were found when comparing CD4+ vs. CD8+ memory cells. The childhood peak for CD4+ memory cells was greater for central-memory cells vs. effector-memory cells [321 cells/µL (95% CI: 269–373) (**Figure 7F**) vs. 212 cells/µL (95% CI: 164–261) (**Figure 7G**)]. The opposite was observed for CD8+ memory cells [61 cells/µL (95% CI: 46–76) for central-memory cells (**Figure 8E**) vs. 282 cells/µL (95% CI: 218–346) for effector-memory cells (**Figure 8F**)]. The peak count for CD8+ effector cells was greater [459 cells/µL (95% CI: 357–561) vs. 343 cells/µL (95% CI: 243–443)] and occurred later (1.0–2.5 years of age vs. 0.5–1.0 year of age) vs. CD4+ counterparts (**Figures 8G**, **7H**).

When examining age-related changes in blood counts for less differentiated T-lymphocytes, maximal counts of CD4+ and CD8+ naïve cells were achieved earlier (3 to 6 months of age) vs. total (6.0 to 12 months of age) and other specific subpopulations. Moreover, differences in peak counts were identified between two phenotypes delineated by the lymph-homing markers CCR7 and CD62L (see **Supplementary Figure 8**). The peak count of CD45RA+ CD62L+ naïve cells was significantly greater than for CD45RA+ CCR7+ naïve cells (2410 cells/µL (95% CI: 2036–2784) vs. 1589 cells/µL (95% CI: 1276–1903) for the CD4+ population; 815 cells/µL (95% CI: 715–916) vs. 467 cells/µL (95% CI: 368–565) for the CD8+ population). Differences between these subpopulations were observed for the first 5 to 10 years of life only; such differences were no longer observed later in life.

Publication bias as well as data heterogeneity were evaluated for each age bin and each subpopulation through a visual inspection of funnel plots and quantification using Egger’s test, as presented in **Supplementary Figures 9**-**25**. When high data heterogeneity was observed (funnel plots asymmetry and Egger’s test with a p-value <0.001) within a selected age bin, further splitting of the bin was tested if the number of measurements for meta-analysis allowed to do so. Publication bias and high heterogeneity in the data was observed for the majority of T-lymphocyte subpopulations in the 0–10 years of age interval. This may be associated with the increase and subsequent sharp decline in cell counts, in the first years of life. Nevertheless, the exclusion of specific observations, along with the aggregation or disaggregation of the age bins associated with observed publication bias, did not result in significant alterations to the weighted means or the loss of significance in Egger’s test.

Comparative results using the selected weighing method (weighing on subject numbers) vs. the inverse variance weighing method are presented in **Supplementary Figures 26**-**28**. For the majority of T-lymphocyte subpopulations, weighted averages calculated by either method were scattered around the identity line. Discrepancies were observed for CD4+ and CD8+ total memory and effector subpopulations. However, greater heterogeneity (as evaluated via Egger’s test) was observed when using the inverse variance weighing method vs. weighing on subject numbers.

## 4 Discussion

Immune aging is a multiplex physiological process characterized by multi-scale dynamic changes at molecular, cellular and tissue levels, such as thymic involution, inflammation, immunosenescence. Our present quantitative analysis of age-related changes covers a wide range of age groups in human, from neonates all the way to centenarians – for the latter, see references (148–151). We did not identify significant age-related differences in the numbers and percentages of immune cells in centenarians, as compared with “younger” adult subjects. Understanding the mechanisms as well as the quantitative evolution of age-related changes in immune cells may help in mitigating the increased susceptibility to infections and tumors and reduced response to vaccination among elderlies. Our analysis shows that peripheral naïve CD4+ and CD8+ T-lymphocyte numbers decreased in mid-life adults and elderlies, as compared to subjects in the 0–10 years-of-age groups - a widely known feature of immune aging, linked to a reduced ability in responding to new antigens (155). Moreover, our meta-analysis did not reveal a substantial decrease in memory T-lymphocyte subsets in elderlies (as compared to mid-life adults), rather a slight increase after ~50 years of age (**Figures 7E–G**, **8D–F**). However, blood numbers of effector CD4+ and CD8+ T-cells tended to decrease with age (**Figures 7H**, **8G**). Coupled with the persistence of immunological memory, this may indicate a reduced potential in the generation of effector cells upon a second antigen encounter (156). Certainly, age-related changes in specific T-lymphocytes, in response to specific antigens need to be evaluated on an individual basis. Age-dependent homeostasis of resident memory cells demonstrated considerable variability (as illustrated in **Supplementary Figures 6**, **7**), with no discernible age-related trends identified among subjects younger than 60 years of age. This finding aligns with the established longevity of these cells and stands in contrast to existing literature that suggests a decline in their presence in blood (157). Nonetheless, the pronounced decrease in resident memory cell counts noted between the age of 20 and 40 warrants further investigation, to elucidate underlying mechanisms.

An interesting outcome of our analysis is the lack of differences in age trends, when comparing T-lymphocyte percentages between blood and lymphoid organs (**Figures 4**, **5**). Since blood counts of immune cells do not always adequately capture the actual immunological status in other organs (158), these meta-analysis derived estimates of cell counts in the blood and lymphoid organs may serve as reference values for healthy subjects, as a starting point to make further comparisons. By comparing rates of decreases in CD4+ and CD8+ T-lymphocytes, we determined that CD8+ T-lymphocytes depleted faster than CD4+ T-lymphocytes, as CD4+/CD8+ ratios tended to increase with age (**Supplementary Figure 5**). In terms of other potential applications, the derived meta-analytical results are not limited to providing such reference values of cell counts in peripheral organs. Indeed, the generalized estimates derived from this study may serve as valuable reference intervals in clinical practice, to assess whether patients are experiencing “healthy” aging. The application of a meta-analytical approach based on a large dataset enabled us to characterize T-cell subpopulations for narrow age intervals, thereby enhancing diagnostic precision. Moreover, researchers may use the proposed findings as historical controls in the design of clinical trials, thereby alleviating the need for a control arm with healthy subjects. Additionally, the proposed methodology – which incorporates specific weighing strategies and handles individual-level data – can be deployed to evaluate age-related dynamics of other cell subsets, under various pathophysiological conditions.

Owing to the large amount of data available in children, we sought to further assess age-related changes within this most variable age group (0 to 5 years of age). Our meta-analysis showed that the age intervals within which blood count peaks were achieved would shift from 3 to 6 months of age to 1 to 5 years of age, moving from less to more differentiated T-lymphocyte subpopulations (**Figures 7**, **8**). Intriguingly, reaching the maximal count for CD4+ RTE T-lymphocytes occurred later than for CD4+ naïve T-lymphocytes (6.0–18 months of age vs. 3.0–6.0 months of age). One possible explanation is that the thymic output may be less efficient than the RTE differentiation into naïve T-cells in infants - coupled with the evidence that RTE cells are long-lived and may remain in the peripheral pool longer than mature naïve cells (159). Alternatively, these data may also be explained by the relatively small amount of CD4+ RTE T-cells in the 0–5 years of age group or, possibly, the non-specificity of the CD31 marker for distinguishing RTE cells as reported recently (158). Also, the selected phenotypes and gating strategies in flow cytometry analyses play a significant role in the determination and quantification of T-lymphocyte subpopulations. As shown for naïve T-cells (**Supplementary Figure 8**), differences in the various markers used may affect cell count data more significantly in younger age groups.

Investigations on quantifying age-dependent homeostasis of lymphocytes have been undertaken by many research groups (25–31). The first attempts to describe such age-related changes of immune cells were made in children. A comprehensive dataset consisting of data from 609 uninfected children (0–7 years of age) born of HIV-infected women was used by Wade and colleagues to construct an exponential model of CD4+ T-lymphocyte blood counts as a function of age (27). Less extensive datasets (smaller number of subjects), yet with greater coverage in terms of various lymphocyte populations have been established to set reference values in healthy children using such an exponential function (26, 28). The most recent work by Schröter and colleagues (25) shed light on age-related changes in lymphocyte numbers in infants (0–1 year of age). These authors showed that blood counts across all considered lymphocyte subpopulations tended to increase in the first year of life. The researchers used a decay exponential function with a constant initial time delay of half a year and an asymptote corresponding to homeostatic levels in healthy adults, to describe age-related changes in lymphocyte blood counts. Our results on maximal counts of the various lymphocyte subsets are in close agreement with those of Schröter et al., with the exception of CD4+ and CD8+ effector cells, whose maximal levels were estimated to be ∼10-fold higher. Such differences may relate to differing gating strategies; for example, effector cells were identified by means of the absence of CD45RO and CD27 markers (25). Moreover, increases in cell counts during the first years of life were observed over longer periods than the initial 6 months of life, for almost all subpopulations under consideration, except for CD4+ naïve T-lymphocytes. Since the model equation proposed by Schröter et al. contained an asymptote with a minimal level of cells to be maintained, the data in the considered range (0 to 62 years of age) were described well. However, dynamic changes in older adults would be difficult to describe using this asymptotic method, since our results show reductions in cell counts for almost all subpopulations. Other investigators quantified age-related changes in lymphocyte counts in adults and elderlies, however sample sizes in these trials were relatively small (29–31).

All model-based analysis approaches discussed above make use of individual-level data – data which are not always readily available. Our meta-analysis approach allowed for the combination and integration of individual- and group-level (or aggregated) data. Handling individual-level data in a meta-analysis is, however, not a trivial task, and several methods have been proposed to account for such data (IPD) (160). We employed a modified two-stage approach: in a first step, average estimates were derived from IPD based on separate studies and within pre-specified age bins; in a second step, the resultant average estimates were combined with originally grouped data, to perform the integrated meta-analysis. We did not perform any regression analysis or analysis of covariance, which are typically used in the first aforementioned stage (160), given the narrow age intervals and the absence of individual characteristic descriptors for subjects. Other approaches, in particular Bayesian approaches, need to be explored to create a more informative combination of individual-level and group-level (aggregated) patient data.

The proposed meta-analysis provided a consistent quantitative description of age-related T-lymphocyte homeostasis across comprehensive datasets; however, it also presents with several limitations. The amount of data relevant to peripheral organs such as kidney, liver and others precluded a quantitative assessment of age-dependent homeostasis in these organs. Also, the determination of age-related changes in stem cell–like memory T-cells (Tscm) as well as regulatory T-cells (Tregs) was out of scope in the present systematic review and corresponding meta-analysis. However, it is the subject of intensive on-going research (161, 162), given the potential of Tscm to maintain T-cell homeostasis and the essential role of Tregs in immunoregulation and autoimmunity development prevention. There is evidence which indicates that CD4+ Tscm counts remain stable, whereas CD8+ Tscm counts tend to decline with age in blood samples (35). The aging process of Tregs appears to be influenced by their origin; specifically, naturally occurring or thymic Tregs seem to accumulate with age, while peripheral or induced Treg populations decline (163). The age-dependent quantification of different types of helper T-cells, especially follicular helper T-cells which mediate antibody production, would also be of interest since the aging of these cells is associated with an increased risk of autoimmune disorders in the elderly population (164). The aging processes of these T-lymphocyte subpopulations deserve a comprehensive investigation that carefully examines and accounts for the various phenotypes associated with these cells. Furthermore, blood counts data in elderly subjects aged >80 years are lacking with respect to several specific T-lymphocyte subpopulations. However, given the existence of relative percentages of specific cell subpopulations (**Figures 4**, **5**), blood numbers for this age range may be calculated. For example, considering naïve CD4+ T-lymphocytes, a reduction in percentage values relative to total CD4+ T-cell concentrations was observed in the >80 years of age group (**Figure 4A**), while the total CD4+ T-lymphocyte count also slightly decreased in that same group (**Figure 7A**). We may thus infer that the blood count (absolute value) of naïve CD4+ T-lymphocytes would also decrease after 80 years of age. However, for other specific subpopulations (central-memory, effector-memory and effector cells), whose percentages increased with age in elderlies (**Figures 4B–D**), trends in blood counts for these cells, coupled with increases in blood counts of total memory cells after ~50 years, may not be intuitive. A model-based meta-analysis may be performed, in future research, to address this specific question.

The proposed analysis investigated the age-related dynamics of various T-lymphocyte subpopulations in all healthy subjects. The influence of external immunological factors, such as CMV infection, on immune aging was not in-scope for this analysis. It is well established that CMV status plays a significant role in accelerating immune aging and modulating the effectiveness of responses to novel infections and vaccinations (165). The presence of CMV infection has been shown to substantially alter the composition of immune cell populations, characterized by a reduction in naïve and an increase in effector-memory and effector subpopulations of both CD4+ and CD8+ T-cells, not only in elderly individuals but also in healthy young adults (63, 85). Furthermore, the CD4+/CD8^+^ ratio exhibits an inversion relative to its non-infected counterparts, with recent studies indicating that this skewing of the T-cell repertoire predominantly occurs in peripheral blood rather than within lymphoid tissues (49, 85). In our systematic review of the literature, five individual studies had been identified, that investigate the impact of CMV status on T-cell subsets and the immune aging process (49, 63, 67, 85, 121). However, we exclusively analyzed data pertaining to CMV-negative subgroups. The remaining data sources lacked information regarding participants’ CMV status. Further exploration of the influence of CMV status on various T-cell subpopulations and their associated aging processes, with a determination of adequate meta-analytic estimates constitutes an important area of future research, for a further characterization of immune aging and individual variability interpretation.

Furthermore, the influence of non-immunological factors, such as sex and ethnicity on immune aging necessitates further investigation. Regarding sex differences and their potential impact on T-cell homeostasis, it remains unclear to what extent such influences are understood. Several studies have been undertaken to explore how subjects’ sex affects the T-lymphocyte repertoire and immune aging (31, 149, 150, 166). The methodologies employed in these studies primarily relied on correlations and linear regression analyses, which impose certain limitations on the interpretation of sex differences. The significance of subjects’ sex on age-related dynamics was assessed through numerical comparisons of p-values of correlation coefficients or of slope parameters across male and female groups. The proposed meta-analytic methodology offers opportunities for a quantitative evaluation and statistical comparison of T-lymphocyte counts across various groups through weighted means comparison or by incorporating covariates. However, not all data sources provided the relevant information to distinguish subjects by sex. We made a concerted effort to include both individual and aggregated data in the curated dataset; for individual subject data, “sex” was not reported in every case, while for aggregated data, “sex” was accounted for as a percentage of male/female subjects whenever this information was provided in the corresponding article. This limited our ability to test “sex” as a categorical covariate and to compare results upon completing the full meta-analysis. Nonetheless, a preliminary analysis on the influence of sex on immune aging was initiated by combining individual data with known subjects’ sex alongside with aggregated data from studies focused on single-sex groups (**Supplementary Figures 29**-**31**). No significant differences were revealed in the age dynamics of total lymphocyte, CD3+ lymphocyte, or CD4+ and CD8+ T-lymphocyte counts, when comparing males vs. females (**Supplementary Figure 29**), in agreement with recent studies (150). Notably, an inversion of the CD4+/CD8+ ratio in older individuals was not observed, contrary to findings reported by (166). Nevertheless, we noted that the magnitude of the CD4+/CD8+ ratio was consistently but not significantly higher among females vs. males, which is also consistent with (166). Furthermore, the data on percentages of specific CD4+ and CD8+ T-lymphocyte subsets across different age groups did not overlap between male and female subjects (in particular, for lymphoid organs), which limited the interpretation of sex differences (**Supplementary Figures 30**-**31**). Given the constraints imposed by the limited data for the first years of life and the lack of consideration of aggregated datasets, expanding our methodology for future investigations remains a priority. One potential strategy to analyze aggregated data that incorporate sex differences in age-related dynamics may involve the use of “sex” as a continuous covariate within each age interval or the use of a non-linear meta-regression.

The proposed analysis focused on flow cytometry data for the main developmental cell populations, for which sufficient data were available. However, the emergence and use of newer technologies for assessing and characterizing immune homeostasis, such as single-cell transcriptomics and multimodal assays, has led to the identification of rare cell populations and enhanced the resolution of immune cell analyses at the single-cell level (167). Given such advantages, these innovative methods have been increasingly used to characterize cell subpopulations that have been previously under-explored through flow cytometry analyses, largely due to limitations associated with existing antibody clones and challenges in labelling intracellular protein expression. Consequently, an integration of data derived from these novel techniques (i.e., scRNA-seq and ATAC-seq) with cytofluorometry data systematically collected in the present study would present conceptual challenges; the marker sets used for cell identification differ between these methodologies. Other design parameters in flow cytometry data analyses include the selection of markers and the gating strategy. The intense development of novel techniques for cell analyses raises questions on the relevance of markers used to identify certain cell subpopulations. As mentioned previously, the use of the CD31 biomarker for distinguishing RTE cells may be questionable, when considering the non-obligatory loss of CD31 marker expression as naïve cells divide in the periphery (168). There are other markers which can be used to distinguish RTE cells which include protein tyrosine kinase 7 (PTK7) and CD103; however, their usage is also limited and remains under debate (3, 169). The PTK7 marker is also highly expressed on naïve T-cells, while the CD103 marker is expressed mostly on CD8+ T-cells. A new distinguishing marker for RTE cells – CD38 – was proposed recently (158); however, a strong correlation between the expression of CD31+ and CD38+ on RTE cells had been reported. Combined with the large amount of cytofluorometry data presented for RTE cells expressing the CD31 marker, it justified the use of this particular marker in the proposed analysis. Another point of debate is the use of the CD69 marker to identify resident-memory T-cells. CD69 plays a dual role as an early activation marker and a marker of tissue residency (170). In the proposed analysis, the subpopulations of CD69+ CD45RO+ expressing CD4+ and CD8+ T-cells were considered as resident memory T-lymphocytes, and their age-dependent homeostasis was explored in peripheral tissues (**Supplementary Figure 6A**). Nonetheless, the proposed meta-analytical methodology can be effectively applied to multi-omic data and cell subpopulations distinguished by other markers, once a sufficient amount of information becomes available in the literature.

In meta-analyses, heterogeneity in the data may be handled through the use of certain weighing strategies, based on subject numbers. We did not apply the commonly used heterogeneity measure I² (or Cochran’s Q test) in the present analysis, due to reliability and stability concerns when based on a limited number of events - an issue exacerbated by our use of age binning (171). A sensitivity analysis was performed to address the variability associated with gating strategies. Regarding lymph homing markers (CCR7 and CD62L), we performed separate meta-analyses based on the selected lymph-homing markers. The only difference detected was for naïve CD4+ and CD8+ cells, especially during the first years of life (as shown in **Supplementary Figure 8**). For other markers used to gate on naïve, central-memory, effector-memory, and effector T-lymphocytes (CD27, CD28), there were limitations in conducting such an analysis due to insufficient amounts of available data for these particular phenotypes. Nevertheless, we undertook a sensitivity analysis comparing weighted means with and without the inclusion of CD28 and/or CD27 markers within the gating strategy for naïve CD4+ and CD8+ T-lymphocytes – since, for these cells, the percentage of phenotypes including CD27/CD28 markers was the highest among other subpopulations (see **Supplementary Table 2**). The sensitivity analysis showed that there was no influence of the CD27/CD28 gating on the generalized estimates across age bins, as illustrated in **Supplementary Figure 32**.

Although the presented meta-analysis provides a quantitative snapshot of immune cell phenotype composition and its age-dependent changes, it does not consider processes of immune differentiation and dynamics. A systems immunology approach to modeling can be applied in order to overcome this limitation and quantitatively assess age-dependent changes in T-lymphocytes circulation and their phenotypic differentiation, in a similar manner to the mechanistic pharmacokinetic and pharmacodynamic modeling actively used in support of drug development (172). For example, quantitative systems pharmacology models, through the integration of biological and patho-physiological mechanisms would enable the community to consistently combine multi-scale experimental data - including pharmacokinetic profiles of therapeutic drug and vaccine treatments - to obtain a mechanistic and quantitative description of the immune system.

## 5 Conclusions

A systematic review and quantitative meta-analysis of data on age-dependent homeostasis focused on T-lymphocyte subpopulations in healthy subjects was performed. An extensive database containing 11,722 unique observations of quantitative measurements for 20 different T-lymphocyte subpopulations from 124 distinct studies was compiled and used in the analysis. The broad coverage of subjects aged from neonates to centenarians enabled us to explore age-related changes in lymphocyte counts in blood and other organs. The generalized estimates of cell counts were calculated for each T-lymphocyte subpopulation of interest, within narrow age intervals. Our analysis showed that the most significant decline in blood counts was observed for total and less differentiated lymphocyte subpopulations within the first 10 years of life. Different subpopulations of memory T-lymphocytes tended to increase with age after 50 years of life. No differences in age-dependent homeostasis were found among relative values of lymphocytes in blood vs. lymphoid organs. Differences in times-to-maximal cell counts were found between less and more differentiated cells, with the latter reaching the maximal number later. The emerging approach of mechanistic model-based meta-analyses, such as the one we presented here allows to systematically explore and quantify various immunological profiles, by maximizing information extraction and exploitation out of large yet heterogeneous experimental and clinical datasets. The proposed approach has been uniquely applied to the systematic exploration of immune aging and the corresponding lifetime dynamics of several key T-lymphocyte subpopulations.

## Data Availability

The original contributions presented in the study are included in the article/**Supplementary Material**. Further inquiries can be directed to the corresponding author.
