# Supplementary material for: Systematic review and quantitative meta-analysis of age-dependent human T-lymphocyte homeostasis
Source: Front Immunol. 2025 Jan 27;16:1475871. doi: 10.3389/fimmu.2025.1475871 (PMC11808020; doi:10.3389/fimmu.2025.1475871)
Supplement: Supplementary file 1 [file DataSheet1.pdf]

Supplementary Material

Systematic review and quantitative meta-analysis of age-dependent human T-lymphocyte homeostasis

Victoria Kulesh[1,2*], Kirill Peskov[1,2,3], Gabriel Helmlinger[4], Gennady Bocharov[2,5,6]

*** Correspondence:** Victoria Kulesh: viktoriaan37@gmail.com

1 Supplementary Figures and Tables

1.1 Supplementary Tables

Supplementary Table 1. Full queries used in the PubMed database searches

**Query**	**Number of entries**
"CD4"[All Fields] AND ("CD62L"[All Fields] OR "CD45RO"[All Fields] OR "CD45RA"[All Fields]) AND ("naive"[All Fields] OR "central-memory"[All Fields] OR "effector-memory"[All Fields] OR "effector"[All Fields]) AND ("human s"[All Fields] OR "humans"[MeSH Terms] OR "humans"[All Fields] OR "human"[All Fields]) AND ("control"[All Fields] OR "healthy"[All Fields]) AND "flow cytometry"[All Fields]	457
("RTE"[All Fields] OR ("recent-thymic"[All Fields] AND ("emigrants and immigrants"[MeSH Terms] OR ("emigrants"[All Fields] AND "immigrants"[All Fields]) OR "emigrants and immigrants"[All Fields] OR "emigrant"[All Fields] OR "emigrants"[All Fields] OR "emigrate"[All Fields] OR "emigrated"[All Fields] OR "emigrates"[All Fields] OR "emigrating"[All Fields] OR "emigration and immigration"[MeSH Terms] OR ("emigration"[All Fields] AND "immigration"[All Fields]) OR "emigration and immigration"[All Fields] OR "emigration"[All Fields] OR "emigrations"[All Fields] OR "emigres"[All Fields]))) AND ("human s"[All Fields] OR "humans"[MeSH Terms] OR "humans"[All Fields] OR "human"[All Fields]) AND ("lymph nodes"[MeSH Terms] OR ("lymph"[All Fields] AND "nodes"[All Fields]) OR "lymph nodes"[All Fields] OR ("lymph"[All Fields] AND "node"[All Fields]) OR "lymph node"[All Fields])	36
("adp ribosyl cyclase 1"[Supplementary Concept] OR "adp ribosyl cyclase 1"[All Fields] OR "cd38"[All Fields] OR "adp ribosyl cyclase 1"[MeSH Terms]) AND "HLADR"[All Fields] AND ("t lymphocytes"[MeSH Terms] OR "t lymphocytes"[All Fields] OR "t lymphocytes"[All Fields]) AND ("lymph nodes"[MeSH Terms] OR ("lymph"[All Fields] AND "nodes"[All Fields]) OR "lymph nodes"[All Fields]) AND ("human s"[All Fields] OR "humans"[MeSH Terms] OR "humans"[All Fields] OR "human"[All Fields])	21

Supplementary Table 2. T-lymphocyte subpopulation phenotypes captured in the database for subsequent meta-analyses

Total subpopulations

Cell type	Essential surface markers	Selected gating strategy captured in the database	% of observations: Grouped Abs.	Grouped Rel.	Individual Abs.	Individual Rel.
**Total Lymphocytes**	CD45+	CD45+ (100%)	5.7	0.3	94.0	0
**Total T-lymphocytes**	CD3+	CD3+ (100%)	8.8	4.2	72.6	14.5
**CD4+/CD8+ ratio**	-	-	10.3	-	89.7	-

Specific subpopulations

Cell type	Essential surface markers	Selected gating strategy captured in the database: CD4+	Selected gating strategy captured in the database: CD8+	% of observations CD4+ Grouped Abs.	CD4+ Grouped Rel.	CD4+ Individual Abs.	CD4+ Individual Rel.	CD8+ Grouped Abs.	CD8+ Grouped Rel.	CD8+ Individual Abs.	CD8+ Individual Rel.
**T-lymphocytes**	CD3+	CD3+ (100%)	CD3+ (100%)	9.3	5.1	66.3	19.3	9.0	5.1	65.5	20.4
**Memory T-lymphocytes**	CD45RA-<br>CD45RO+	CD45RO+ (91.78%)<br>CD45RA- (7.04%)<br>CD45RO+CD45RA- (0.94%)<br>CD45RO+CD28+ (0.23%)	CD45RO+ (91.73%)<br>CD45RA- (7.49%)<br>CD45RO+CD45RA- (0.52%)<br>CD45RO+CD28+ (0.26)	6.1	6.8	50.2	36.9	5.4	3.4	57.9	33.3
**Recent thymic emigrants (RTE)**	CD31+<br>CD45RA+<br>(1)	CD31+ (23.57%)<br>CD31+ CD25- (56.63%)<br>CD31+CD45RA+ (19.8%)	CR2+CD31+ (99.44%)<br>CD31+ (0.28%)<br>CD31+CD45RA+ (0.28%)	1.8	15.9	13.4	68.9	0	0.6	0	99.4
**Naïve (N) T-lymphocytes**	CD45RA+<br>CD45RO-<br>CD62L+<br>CCR7+<br>(2)	CD45RA+CCR7+ (71.91%)<br>CD45RA+CD45RO-CCR7+ (0.39%)<br>CD45RA+CD62L+ (13.02%)<br>CD45RO-CCR7+ (2.24%)<br>CD45RO-CD27+CCR7+ (0.10%)<br>CD45RA+CD27+CCR7+ (0.39%)<br>CD45RO-CD28+CD95-CCR7+ (8.94%)<br>CD45RO-CD62L+ (0.19%)<br>CD45RA+CD197+ (2.82%)	CD45RA+CCR7+ (74.63%)<br>CD45RA+CD45RO-CCR7+ (0.37%)<br>CD45RA+CD62L+ (9.75%)<br>CD45RA+CD197+ (3.00%)<br>CD45RO-CD27+CCR7+ (0.50%)<br>CD45RA+CD27+CD28+CCR7+ (0.25%)<br>CD45RO-CD28+CD95-CCR7+ (11.5%)	4.4	5.0	14.1	76.5	4.9	5.9	15.1	74.1
**Activated (Act) T-lymphocytes**[*1]	CD38+<br>HLADR+<br>(2)	CD38+ HLADR+ (100%)	CD38+ HLADR+ (100%)	39.5	60.5	0	0	45.5	48.5	0	6.0
**Central-memory (CM) T-lymphocytes**	CD45RA-<br>CD45RO+<br>CD62L+<br>CCR7+<br>(2)	CD45RO+CCR7+ (4.24%)<br>CD45RO+CD197+ (0.15%)<br>CD45RO+CD62L+ (0.30%)<br>CD45RA-CCR7+ (82.42%)<br>CD45RA-CD197+ (4.40%)<br>CD45RA-CD62L+ (6.82%)	CD45RO+CCR7+ (0.81%)<br>CD45RO+CD197+ (0.16%)<br>CD45RO+CD62L+ (0.32%)<br>CD45RA-CCR7+ (85.92%)<br>CD45RA-CD197+ (3.88%)<br>CD45RA-CD62L+ (7.44%)	5.3	7.7	0.8	86.2	4.7	7.6	0.8	86.9

		CD45RO+CD27+CCR7+ (0.15%)<br>CD45RA-CD27+CCR7+ (0.61%)<br>CD45RO+CD45RA-CCR7+ (0.76%)<br>CD45RA-CD27+CD28+CCR7+ (0.15%)	CD45RO+CD45RA-CCR7+ (0.65%)<br>CD45RA-CD27+CCR7+ (0.65%)<br>CD45RA-CD27+CD28+CCR7+ (0.16%)								
**Effector-memory (EM) T-lymphocytes**	CD45RA-<br>CD45RO+<br>CD62L-<br>CCR7-<br>(2)	CD45RO+CCR7- (4.32%)<br>CD45RO+CD197- (0.15%)<br>CD45RO+CD62L- (0.46%)<br>CD45RA-CCR7- (82.25%)<br>CD45RA-CD197- (4.48%)<br>CD45RA-CD62L- (6.79%)<br>CD45RO+CD45RA-CCR7- (0.77%)<br>CD45RO+CD27-CCR7- (0.15%)<br>CD45RA-CD27+CCR7- (0.62%)	CD45RO+CCR7- (0.65%)<br>CD45RO+CD197- (0.13%)<br>CD45RO+CD62L- (0.26%)<br>CD45RA-CCR7- (77.08%)<br>CD45RA-CD197- (3.26%)<br>CD45RA-CD62L- (17.45%)<br>CD45RO+CD45RA-CCR7- (0.52%)<br>CD45RA-CD27+CCR7- (0.52%)<br>CD45RA-CD27+CD28+CCR7- (0.13%)	5.4	7.8	0.8	86.0	3.8	6.2	0.7	89.3
**Effector (EFF) T-lymphocytes**	CD45RA+<br>CD45RO-<br>CD62L-<br>CCR7-<br>(2)	CD45RA+CCR7- (86.28%)<br>CD45RA+CD197- (4.13%)<br>CD45RA+CD62L- (7.44%)<br>CD45RO-CCR7- (0.50%)<br>CD45RA+CD45RO-CCR7- (0.66%)<br>CD45RA+CD27-CCR7- (0.66%)<br>CD45RA+CD27-CD28-CCR7- (0.33%)	CD45RA+CCR7- (76.61%)<br>CD45RA+CD197- (3.42%)<br>CD45RA+CD62L- (19.01%)<br>CD45RO-CCR7- (0.14%)<br>CD45RA+CD45RO-CCR7- (0.55%)<br>CD45RA+CD27-CD28-CCR7- (0.27%)	5.8	7.3	0.8	86.1	3.8	5.6	0.7	89.9
**Resident-memory (RM) T-lymphocytes** *2	CD45RO+<br>CD69+<br>CD103+<br>(3)	CD45RO+CD69+ (66.34%)<br>CD45RO+CD103+ (33.66%)	CD45RO+CD69+ (66.5%)<br>CD45RO+CD103+ (33.5%)	0	0.5	0	99.5	0	0.5	0	99.5

Abs. – Absolute values (units: cells/µL, cells, cells/g, cells/mm²); Rel. – Relative values (units: %)

*1 the database contained data for CD38+ HLADR- and CD38- HLADR+ cells, which were not included in the analysis

*2 the database contained data for CD45RA+ CD69+ and CD45RA+ CD103+ cells, which were not included in the analysis

Supplementary Table 3. Data binning strategy for T-lymphocyte subpopulations

T-lymphocyte subpopulation	Number of bins	Bin age limits
Total lymphocytes	15	0, 0.25, 0.5, 1.0, 2.5, 5.0, 10, 20, 30, 40, 60, 70, 80, 90, 100, 115
Total CD3+ T-lymphocytes	12	0, 0.25, 0.5, 1.0, 2.5, 5.0, 10, 20, 30, 40, 60, 80, 115
Total CD4+ T-lymphocytes	16	0, 0.25, 0.5, 1.0, 2.5, 5.0, 10, 15, 30, 35, 40, 50, 60, 70, 80, 100, 115
CD4+ RTE T-lymphocytes	10	0, 0.5, 1.5, 5.0, 10, 20, 30, 40, 50, 60, 70
CD4+ naïve T-lymphocytes	13	0, 0.25, 0.5, 2.5, 5.0, 10, 20, 30, 40, 50, 60, 70, 80, 90
CD4+ activated T-lymphocytes	7	0, 0.5, 2.5, 10, 20, 40, 60, 80
CD4+ total memory T-lymphocytes	12	0, 0.5, 1.0, 2.5, 5.0, 10, 25, 40, 50, 60, 65, 70, 90
CD4+ central-memory T-lymphocytes	11	0, 0.25, 0.5, 1.0, 2.5, 10, 20, 30, 40, 50, 60, 80
CD4+ effector-memory T-lymphocytes	10	0, 0.25, 0.5, 1.0, 2.5, 10, 25, 30, 40, 55, 80
CD4+ effector T-lymphocytes	9	0, 0.5, 1.0, 2.5, 10, 25, 40, 50, 60, 80
Total CD8+ T-lymphocytes	15	0, 0.25, 0.5, 1.0, 2.5, 5.0, 10, 15, 30, 40, 50, 60, 70, 80, 100, 115
CD8+ naïve T-lymphocytes	13	0, 0.25, 0.5, 2.5, 5.0, 10, 20, 30, 40, 50, 60, 70, 80, 90
CD8+ activated T-lymphocytes	7	0, 1.0, 5.0, 10, 20, 40, 60, 80
CD8+ total memory T-lymphocytes	13	0, 0.5, 1.0, 2.5, 5.0, 10, 20, 30, 40, 50, 60, 65, 70, 90
CD8+ central-memory T-lymphocytes	11	0, 0.25, 0.5, 1.0, 2.5, 10, 25, 35, 40, 50, 60, 80
CD8+ effector-memory T-lymphocytes	11	0, 0.25, 0.5, 1.0, 5.0, 10, 25, 35, 40, 50, 60, 80
CD8+ effector T-lymphocytes	9	0, 0.25, 0.5, 1.0, 2.5, 10, 25, 40, 50, 80

1.2 Supplementary Figures

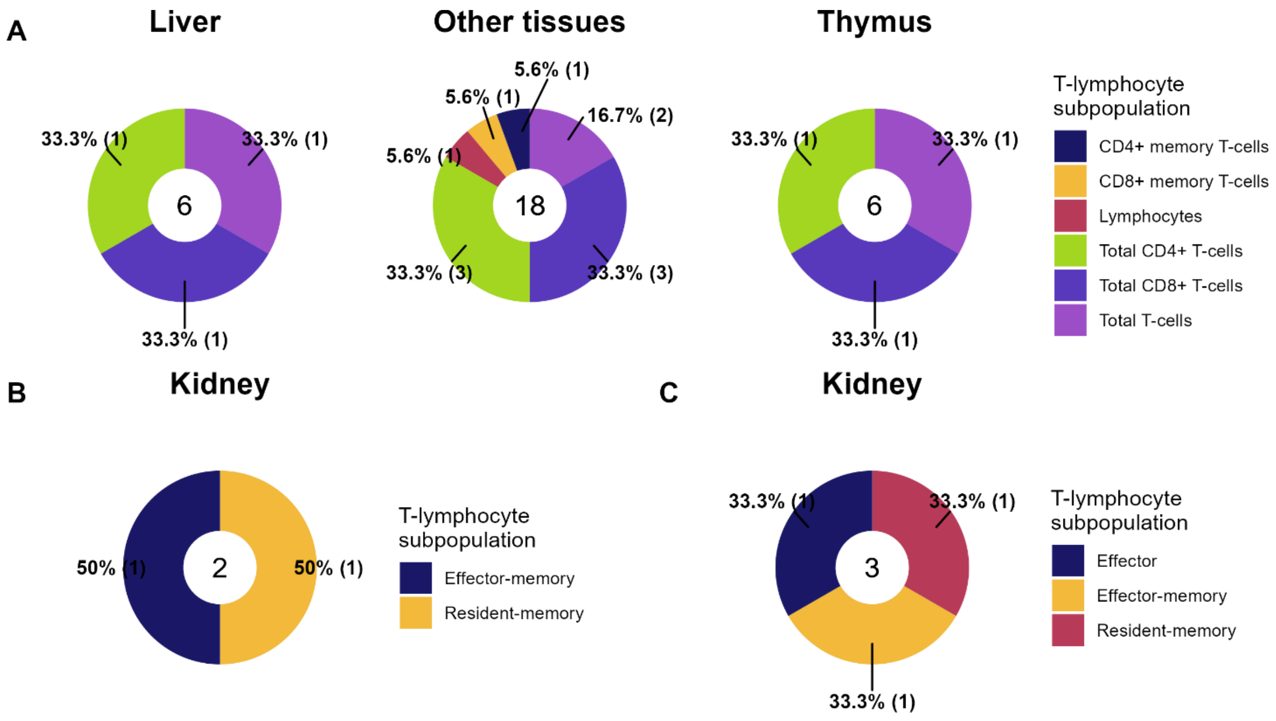

Supplementary Figure 1. Percentage distributions of unique observations for total (A), specific CD4+ (B), and specific CD8+ (C) T-lymphocyte subpopulations in the database across peripheral sites (Kidney, Liver, Thymus, Other tissues). Numbers within donut charts represent the total numbers of observations. The proportion of each T-lymphocyte subpopulation in an organ is provided as a percentage, with the number of unique studies given within the brackets.

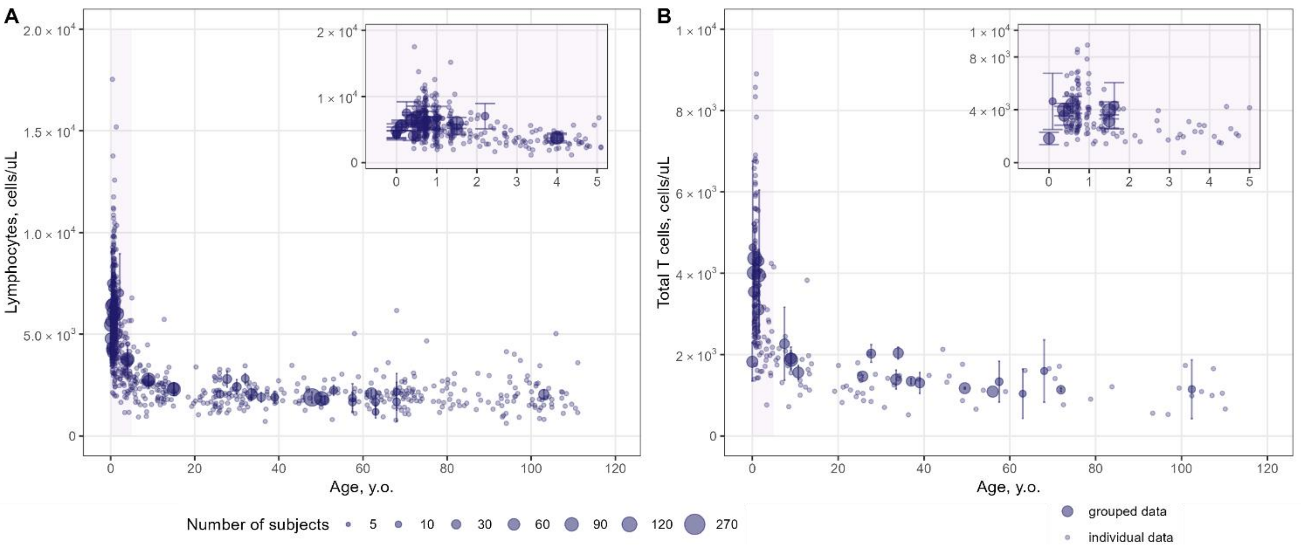

Supplementary Figure 2. Age-dependent homeostasis of total T-lymphocyte subpopulations in blood, expressed in absolute values: A – total lymphocytes (studies: 21; observations: 749); B – total CD3+ T-lymphocytes (studies: 23; observations: 482). Dots represent individual data; dots with error bars represent the means with 95% CIs of grouped data; dot diameters indicate subject numbers in groups; purple shaded areas represent data for neonates, infants and toddlers (0 to 5 years of age).

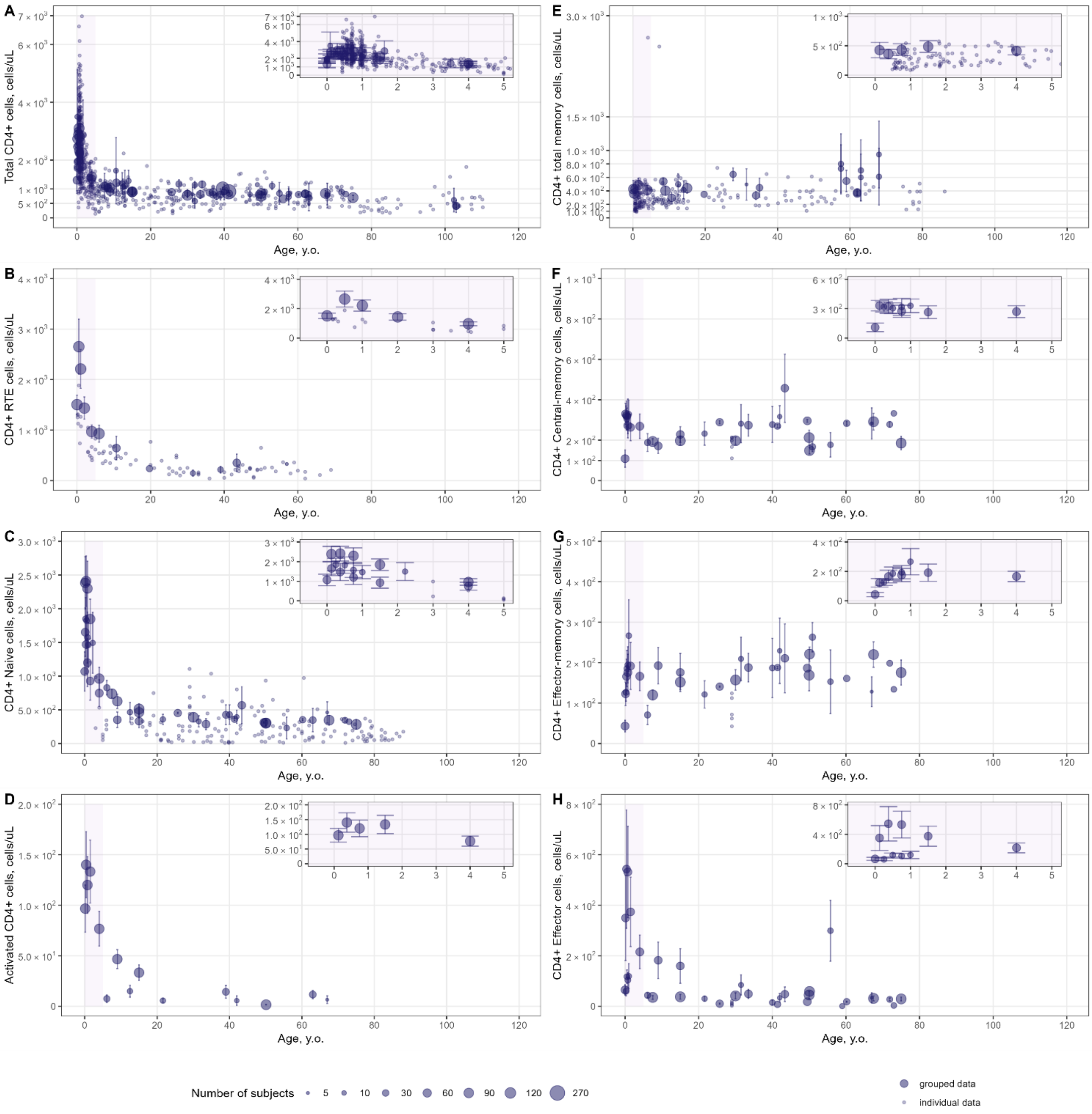

Supplementary Figure 3. Age-dependent homeostasis of CD4+ T-lymphocyte subpopulations in blood, expressed in absolute values: A – total CD4+ (studies: 46; observations: 695); B – CD4+ RTE (studies: 8; observations: 93); C – CD4+ naïve (studies: 19; observations: 190); D – CD4+ activated (studies: 5; observations: 15); E – CD4+ total memory (studies: 15; observations: 240); F – CD4+ central-memory (studies: 13; observations: 40); G – CD4+ effector-memory (studies: 13; observations: 40); H – CD4+ effector T-lymphocytes (studies: 13; observations: 40). Dots represent

individual data; dots with error bars represent the means with 95% CIs of grouped data; dot diameters indicate subject numbers in groups; purple shaded areas represent data for neonates, infants and toddlers (0 to 5 years of age).

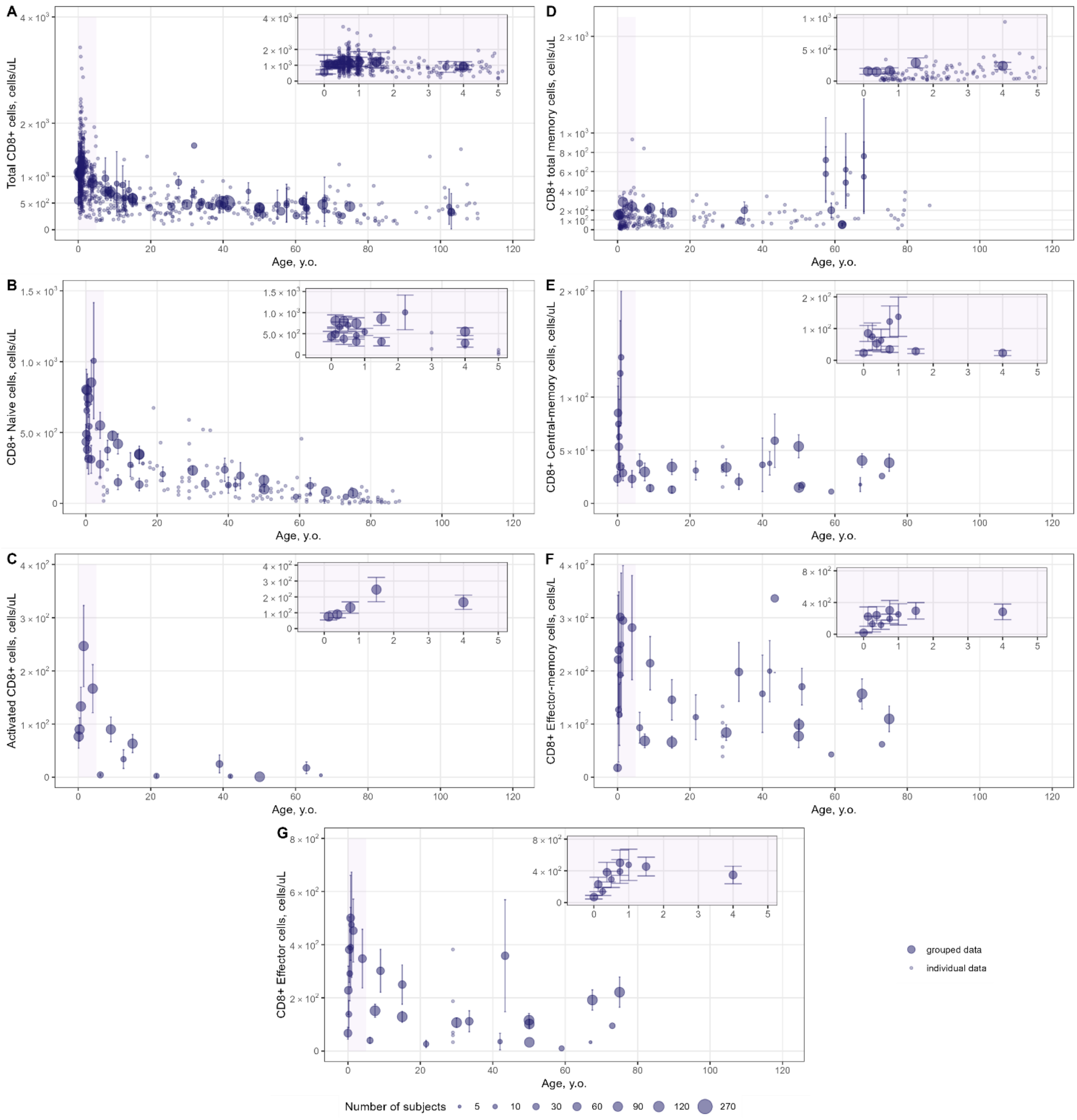

Supplementary Figure 4. Age-dependent homeostasis of CD8+ T-lymphocyte subpopulations in blood, expressed in absolute values: A – total CD8+ (studies: 40; observations: 647); B – CD8+ naïve (studies: 16; observations: 160); C – CD8+ activated (studies: 5; observations: 15); D – CD8+ total memory (studies: 10; observations: 245); E – CD8+ central-memory (studies: 11; observations: 34); F – CD8+ effector-memory (studies: 11; observations: 34); G – CD8+ effector T-lymphocytes

(studies: 9; observations: 33). Dots represent individual data; dots with error bars represent the means with 95% CIs of grouped data; dot diameters indicate subject numbers in groups; purple shaded areas represent data for neonates, infants and toddlers (0 to 5 years of age).

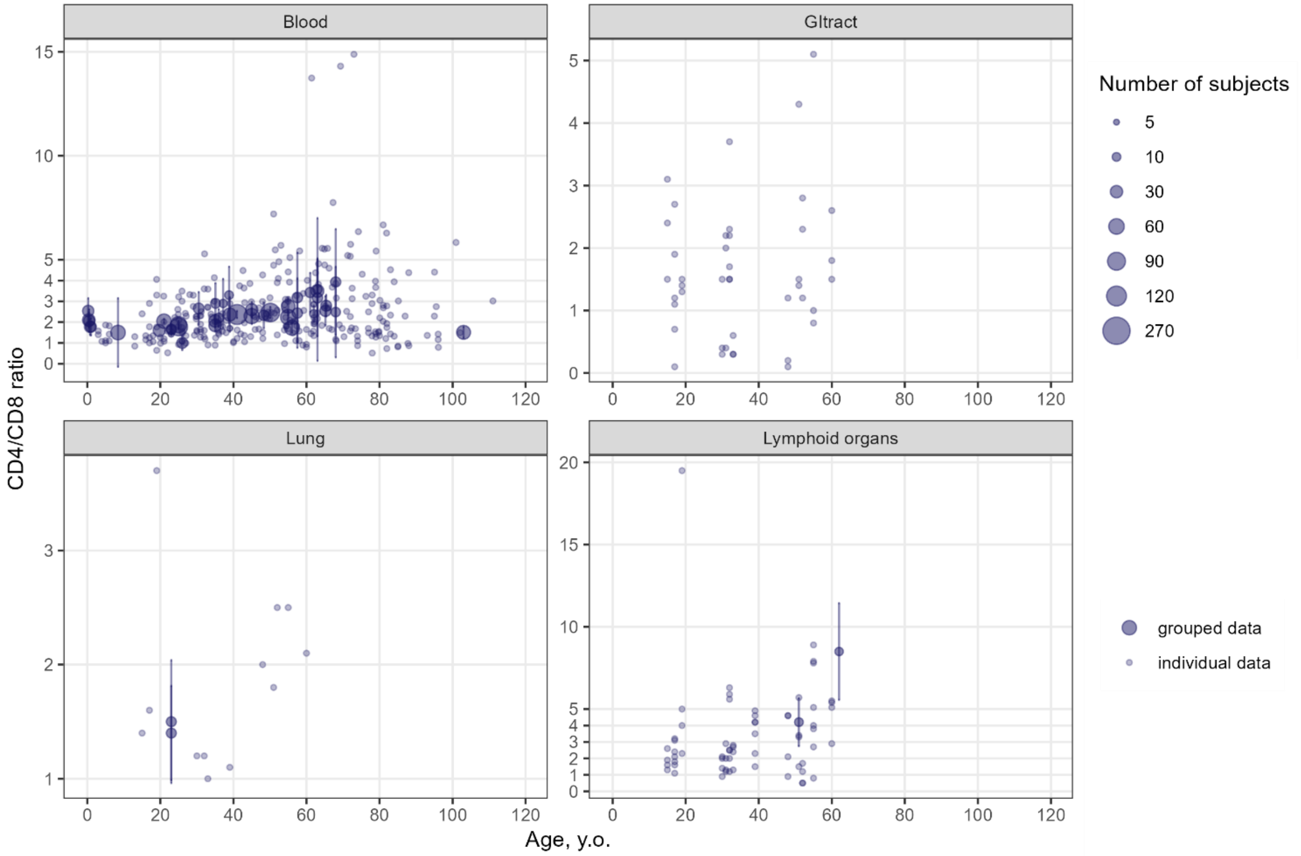

Supplementary Figure 5. Age-dependent homeostasis of CD4+/CD8+ ratios across 4 physiological organs (Blood (studies: 25; observations: 312), GI tract (studies: 1; observations: 42), Lung (studies: 2; observations: 14), Lymphoid organs (studies: 3; observations: 67)). Dots represent individual data; dots with error bars represent the means with 95% CIs of grouped data; dot diameters indicate subject numbers in groups.

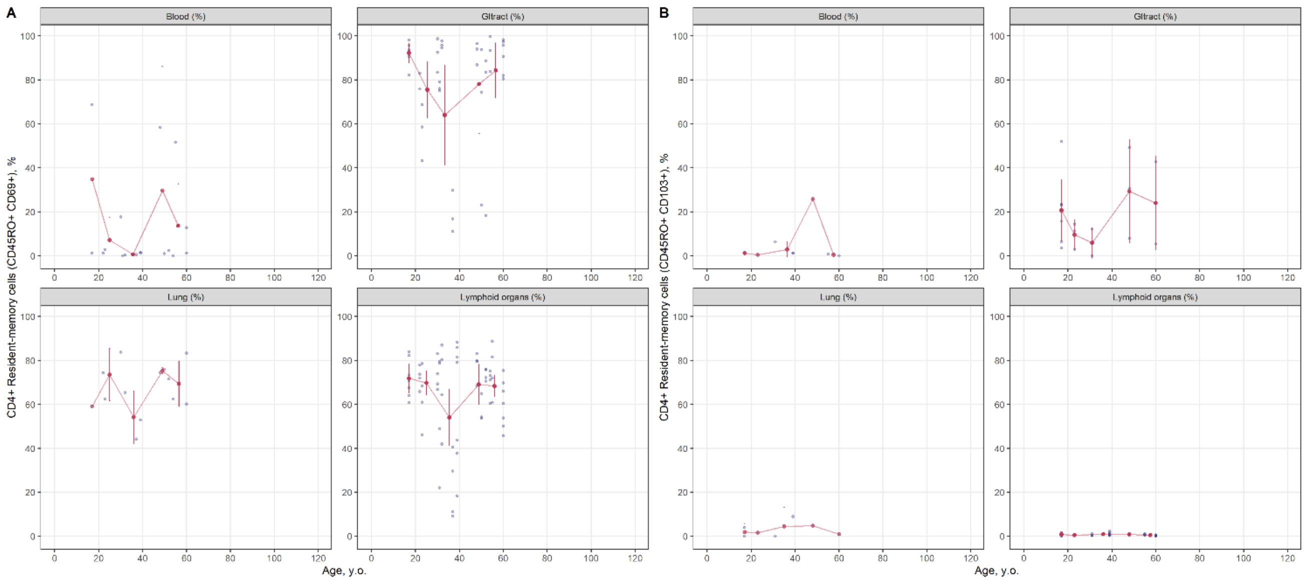

Supplementary Figure 6. Age-dependent homeostasis of CD4+ resident-memory T-lymphocytes across 4 physiological organs. Values are expressed relative to total CD4+ memory T-lymphocytes. A – CD45RO+CD69+ cells (studies: 1); B – CD45RO+CD103+ cells (studies: 1). Dots represent individual data, dots with error bars represent the mean with 95% CIs of grouped data, dot diameters indicate subject numbers in groups; red lines with error bars represent moving averages calculated on individual data with a 10-year step.

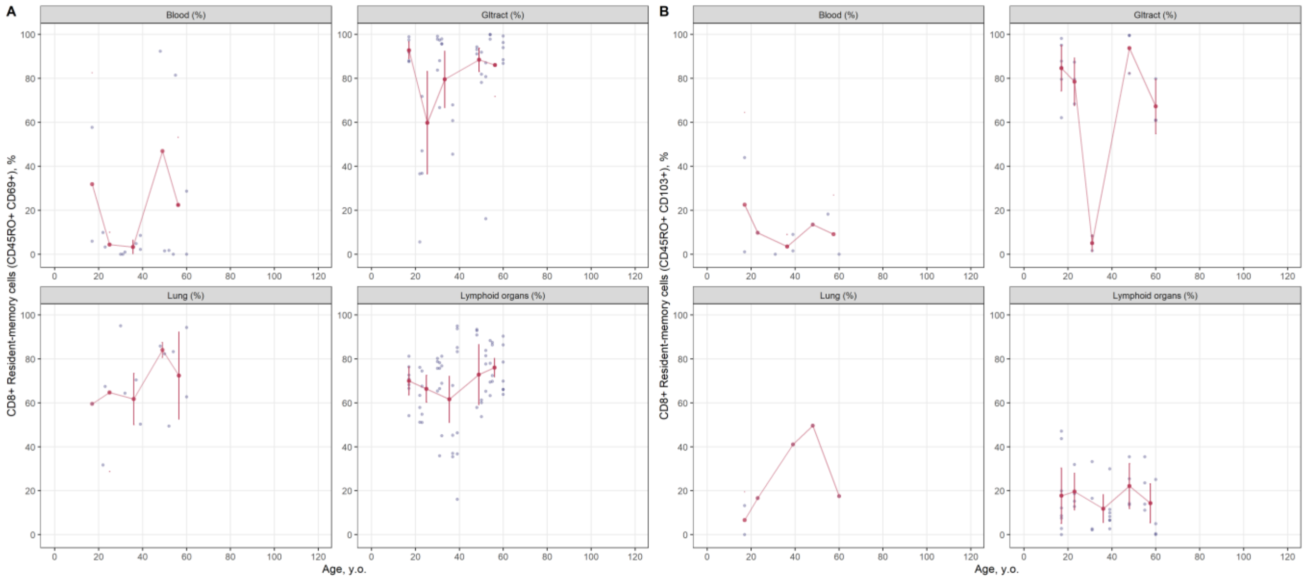

Supplementary Figure 7. Age-dependent homeostasis of CD8+ resident-memory T-lymphocytes across 4 physiological organs. Values are expressed relative to total CD8+ memory T-lymphocytes. A – CD45RO+CD69+ cells (studies: 1); B – CD45RO+CD103+ cells (studies: 1). Dots represent individual data, dots with error bars represent the mean with 95% CIs of grouped data, dot diameters indicate subject numbers in groups; red lines with error bars represent moving averages calculated on individual data with a 10-year step.

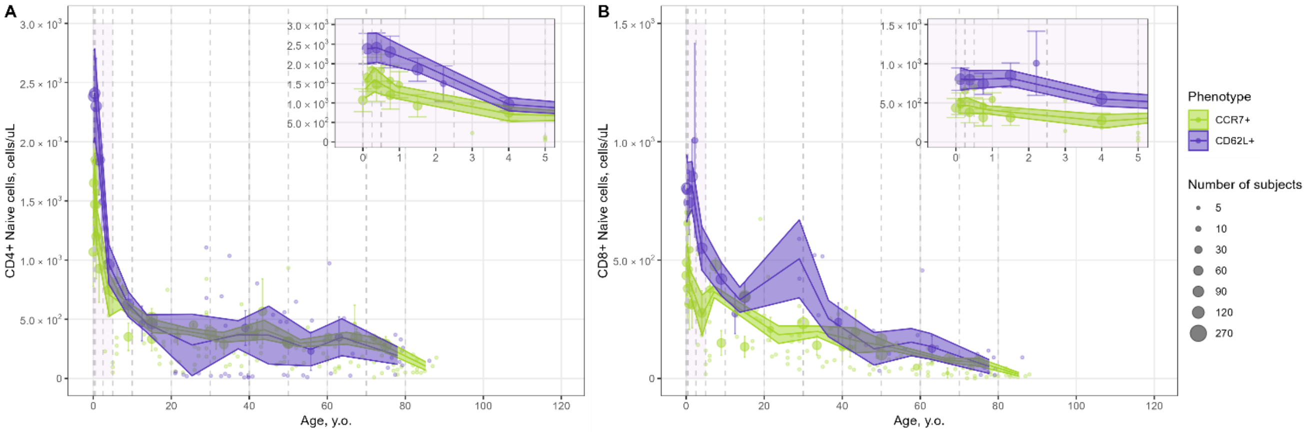

Supplementary Figure 8. Meta-analysis of CD4+ (A) and CD8+ (B) naïve T-lymphocyte age-dependent homeostasis in blood (expressed in absolute values) and depending on cell phenotype used (CD4: CCR7+ (studies: 12; observations: 130); CD62L+ (studies: 7; observations: 60); CD8: CCR7+ (studies: 11; observations: 125); CD62L+ (studies: 5; observations: 35)). Dots represent individual-level data; dots with error bars represent means with 95% CIs of the grouped data; solid lines with shaded areas represent weighted means with 95% CI for each age bin, with bin limits indicated by vertical dashed lines; dot diameters indicate subject numbers in groups; purple shaded areas represent data for neonates, infants and toddlers (0 to 5 years of age).

For Supplementary Figures 9-25: The large white triangles represent the region where 95% of the data points should lie in the absence of publication bias. The vertical dashed line represents the meta-analysis mean. Funnel plot asymmetry was detected using Egger's test. Blue dots: aggregated data; yellow dots: moving average calculated based on individual-level data.

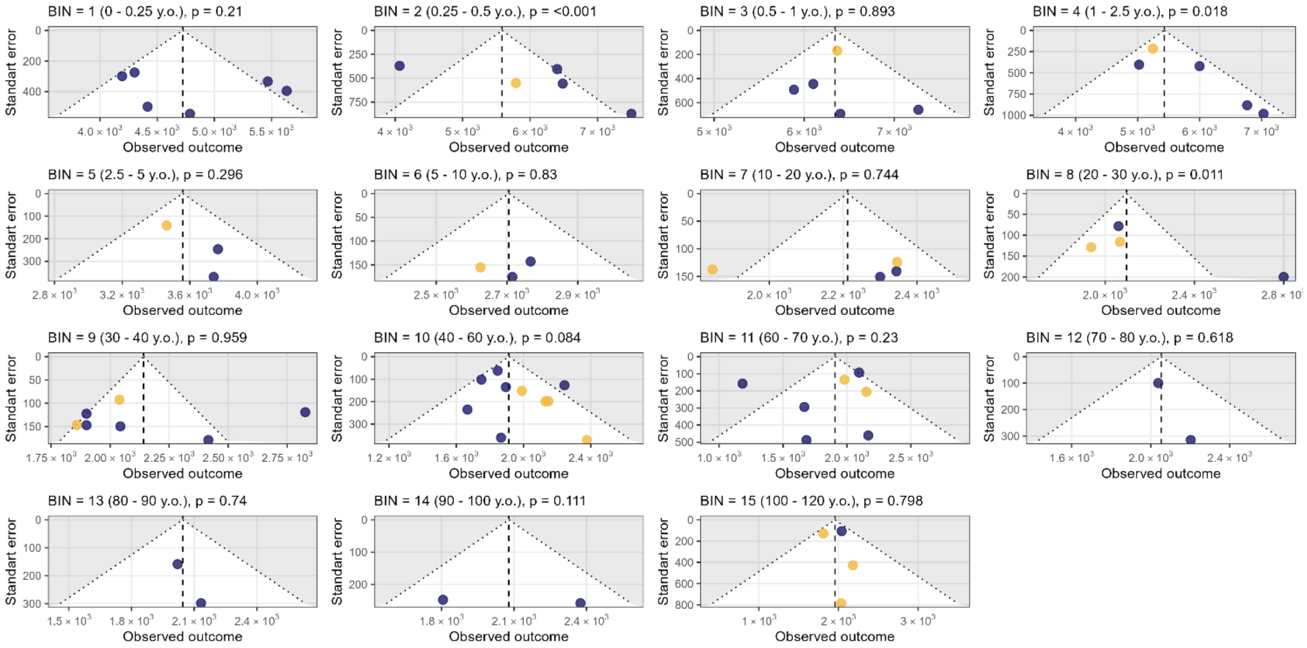

Supplementary Figure 9. Funnel plots for the total lymphocyte subpopulation.

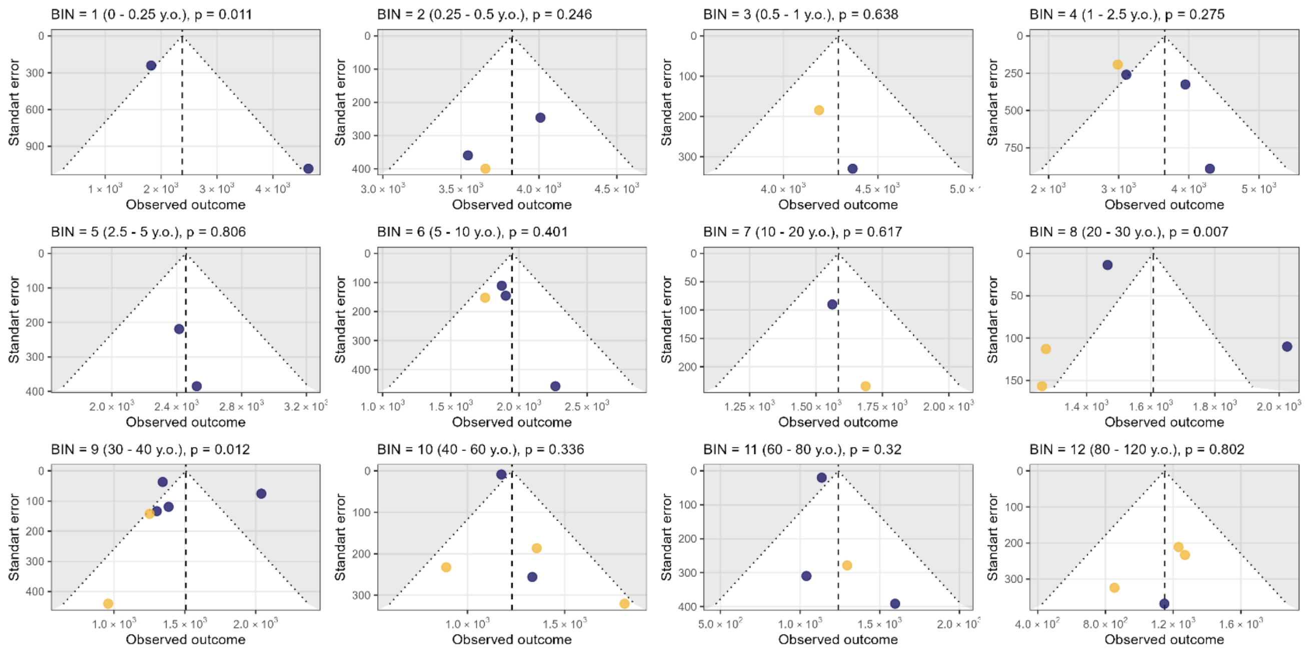

Supplementary Figure 10. Funnel plots for the total CD3+ T-lymphocyte subpopulation.

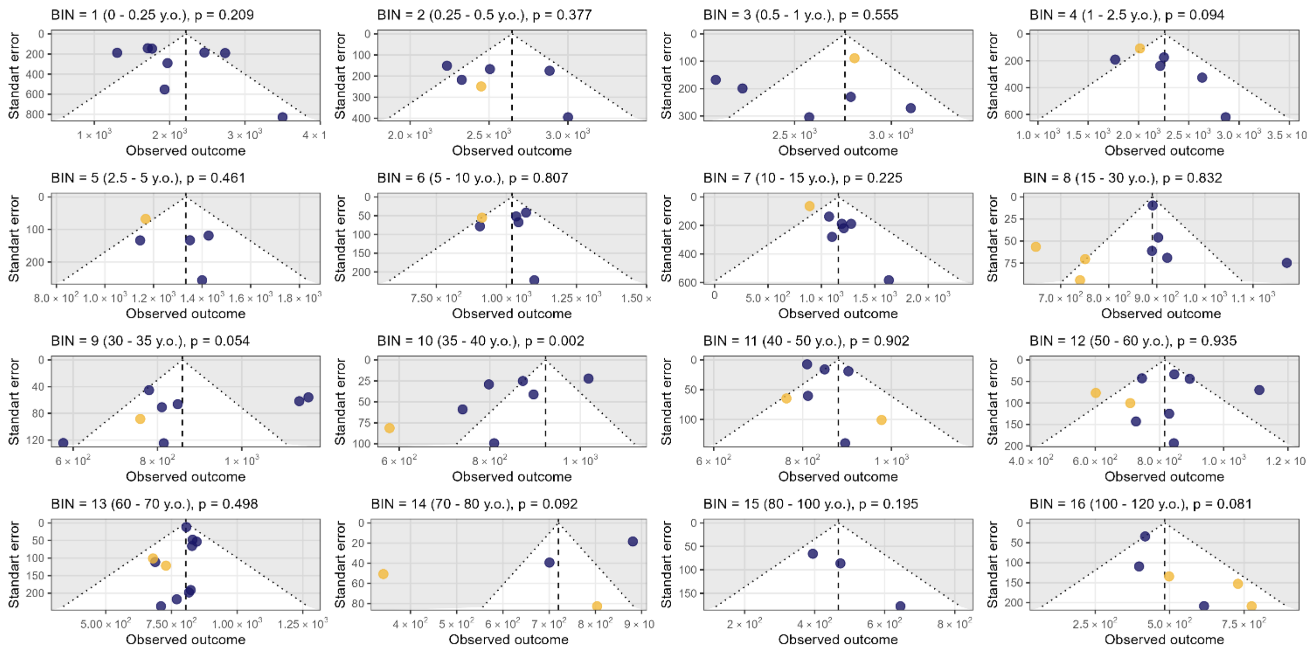

Supplementary Figure 11. Funnel plots for the total CD4+ T-lymphocyte subpopulation.

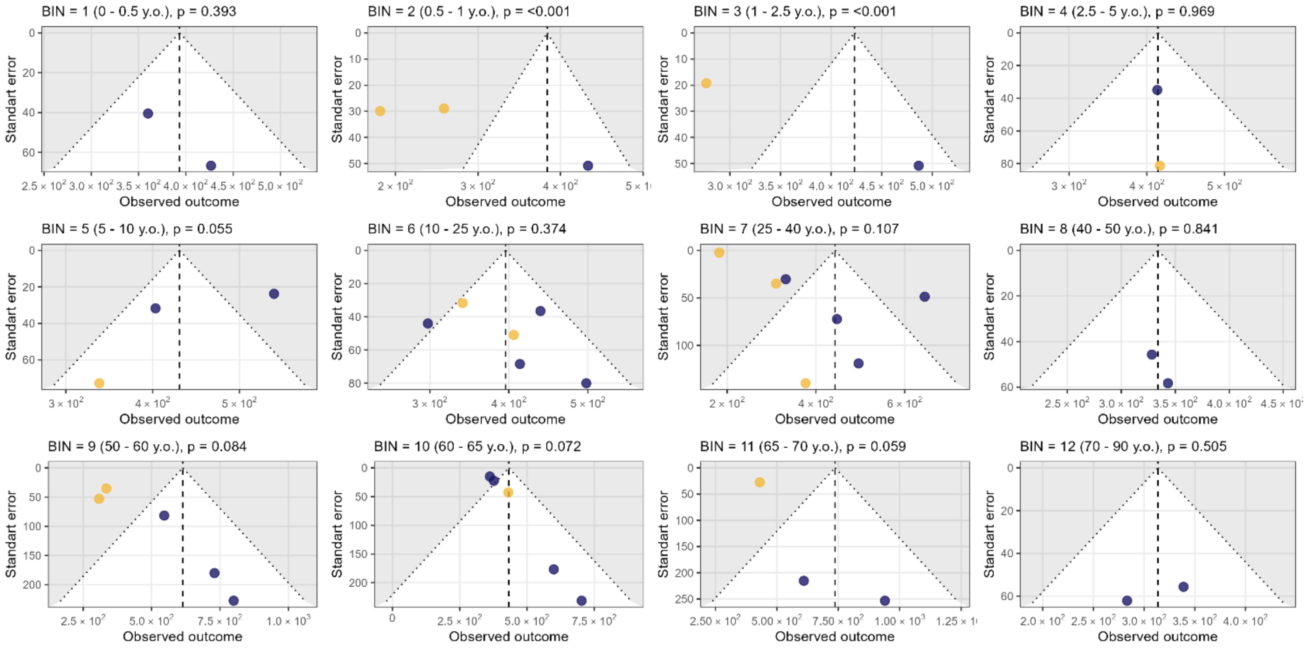

Supplementary Figure 12. Funnel plots for the total memory CD4+ T-lymphocyte subpopulation.

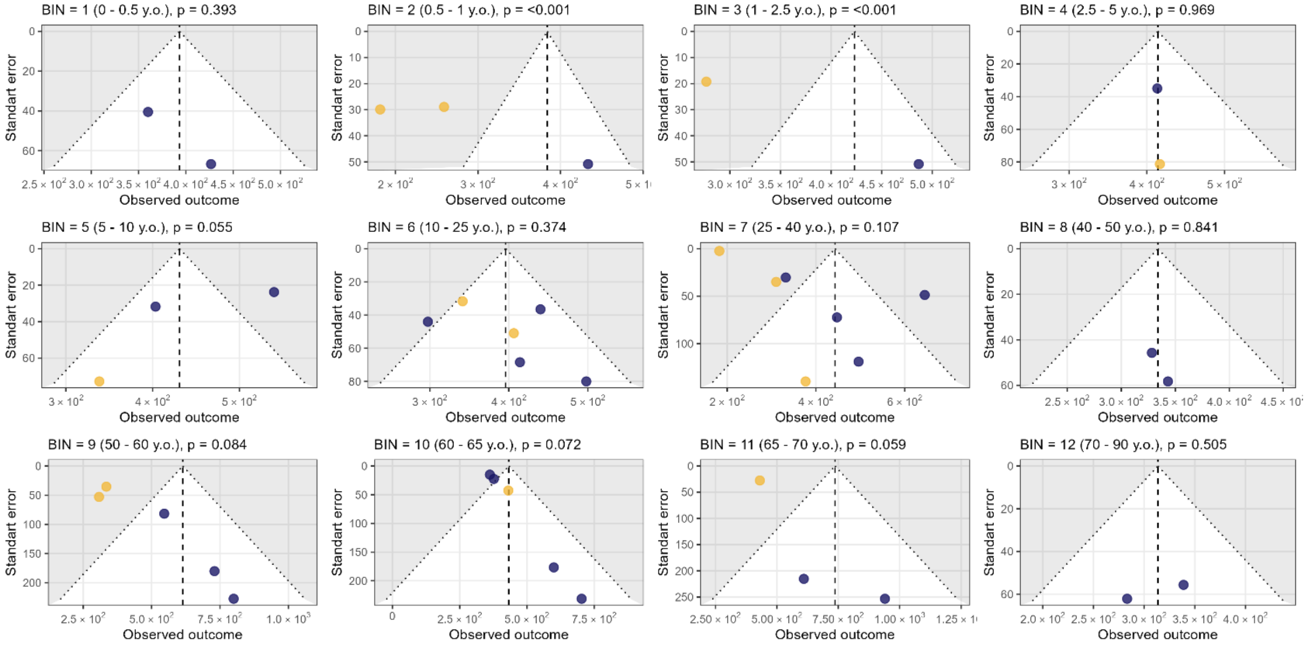

Supplementary Figure 13. Funnel plots for the CD4+ RTE T-lymphocyte subpopulation.

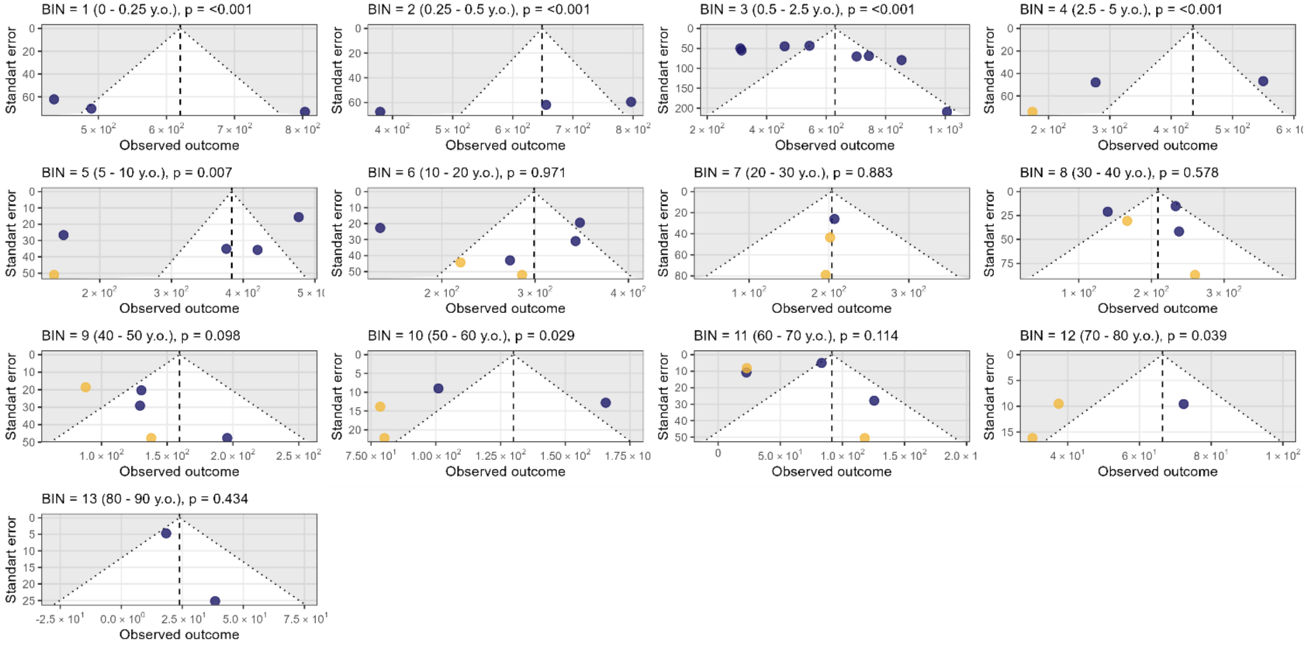

Supplementary Figure 14. Funnel plots for the CD4+ naive T-lymphocyte subpopulation.

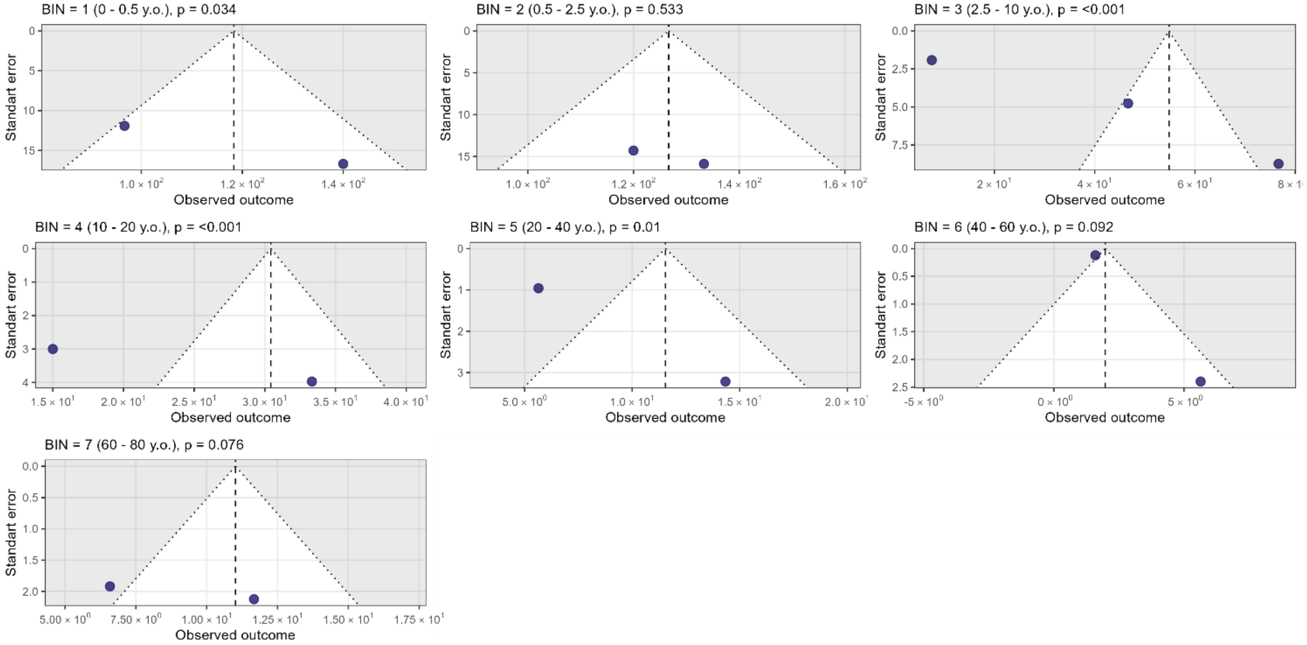

Supplementary Figure 15. Funnel plots for the CD4+ activated T-lymphocyte subpopulation.

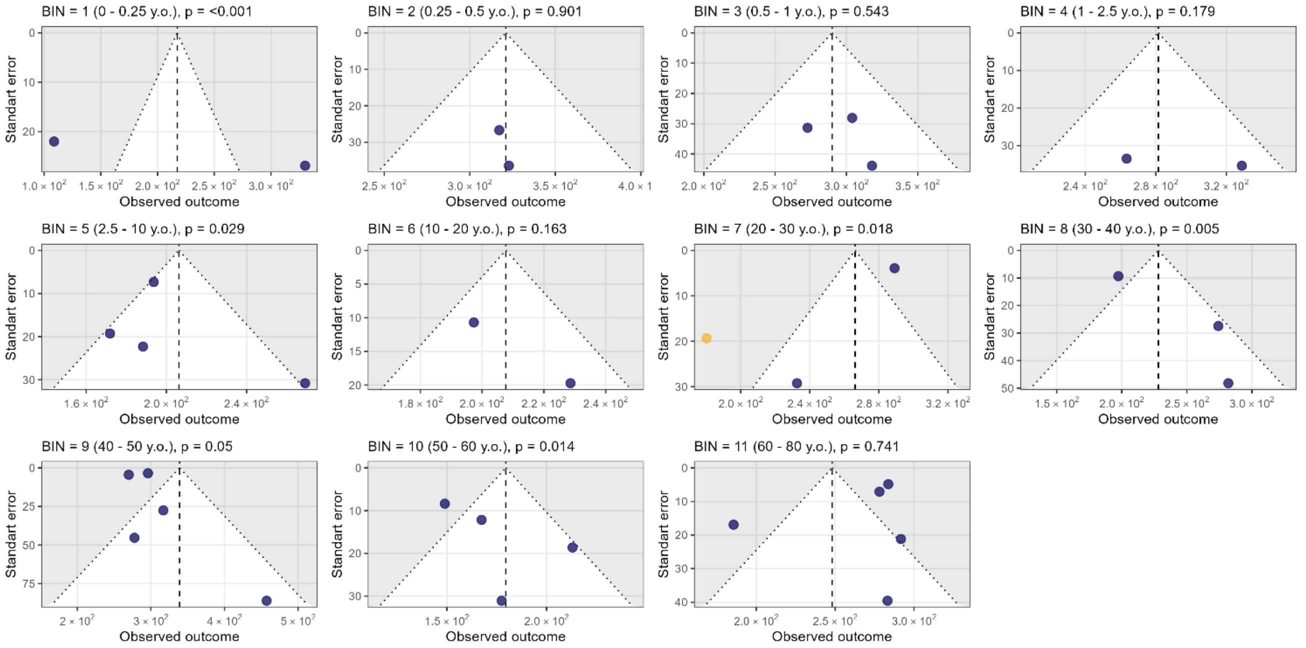

Supplementary Figure 16. Funnel plots for the CD4+ central-memory T-lymphocyte subpopulation.

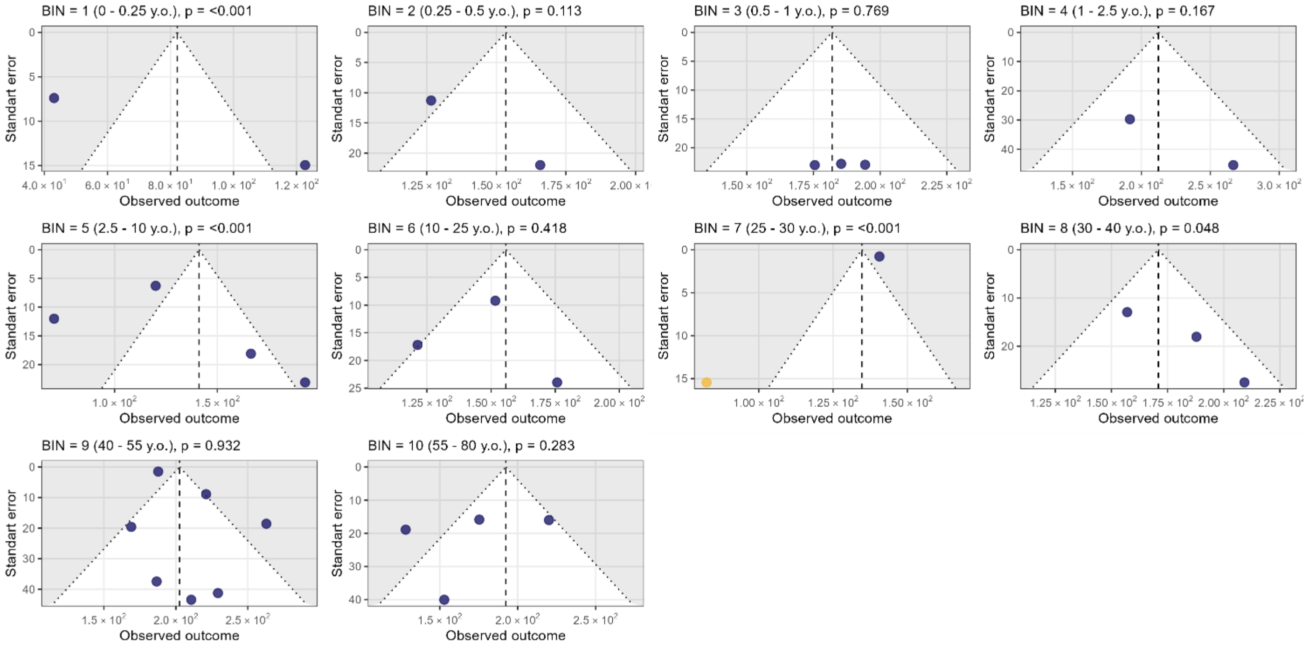

Supplementary Figure 17. Funnel plots for the CD4+ effector-memory T-lymphocyte subpopulation.

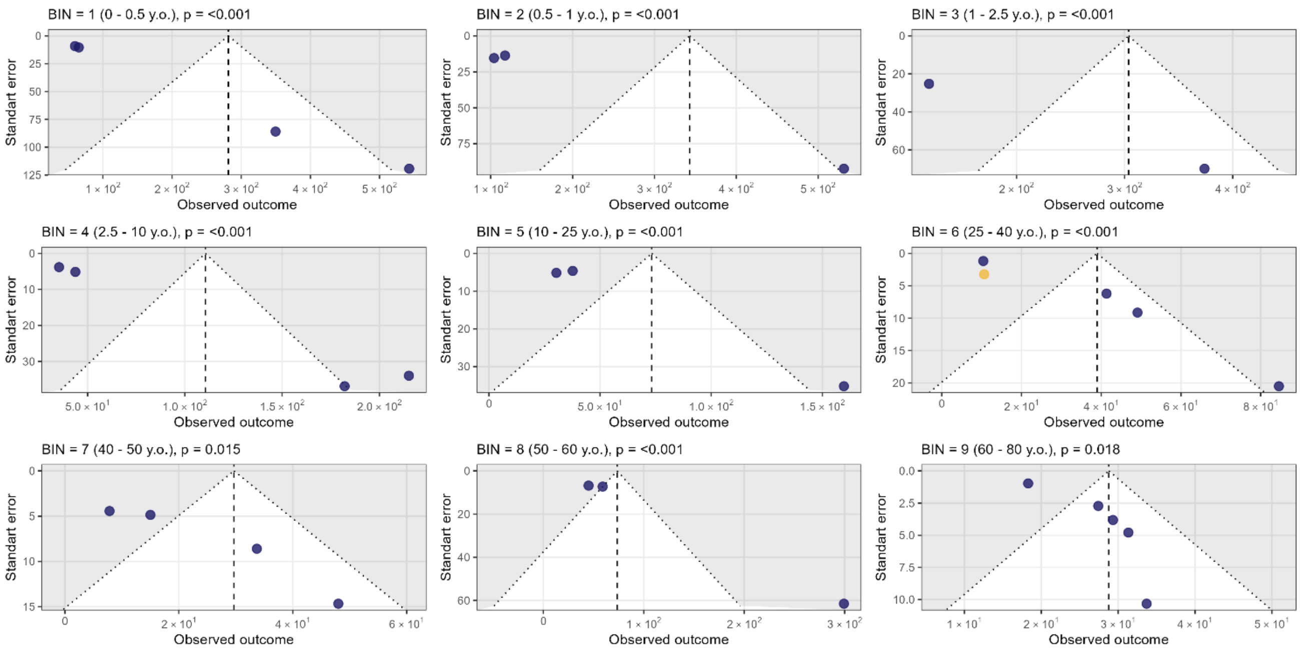

Supplementary Figure 18. Funnel plots for the CD4+ effector T-lymphocyte subpopulation.

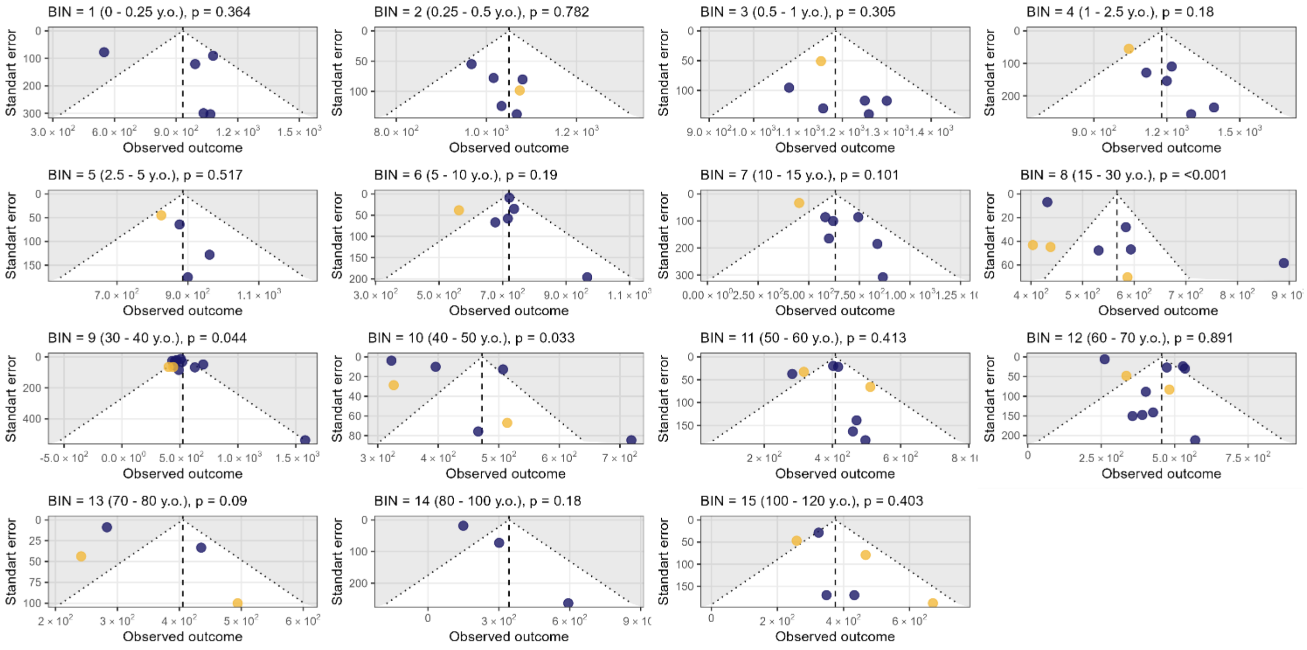

Supplementary Figure 19. Funnel plots for the total CD8+ T-lymphocyte subpopulation.

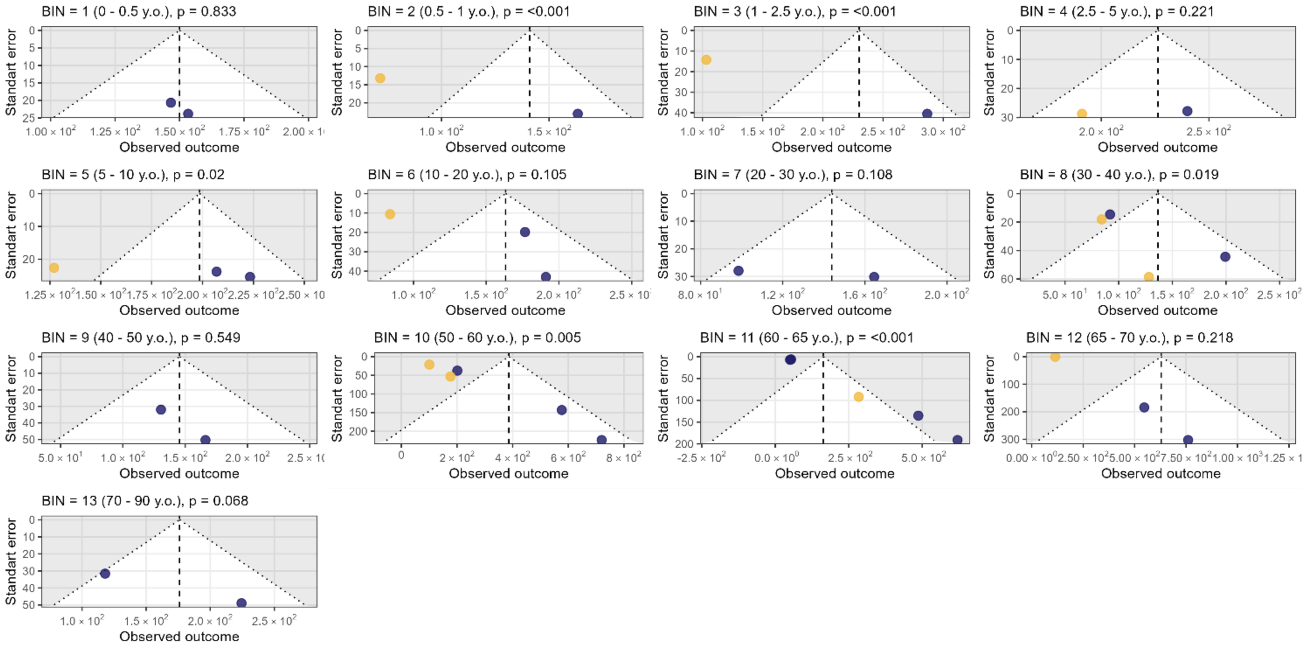

Supplementary Figure 20. Funnel plots for the total memory CD8+ T-lymphocyte subpopulation.

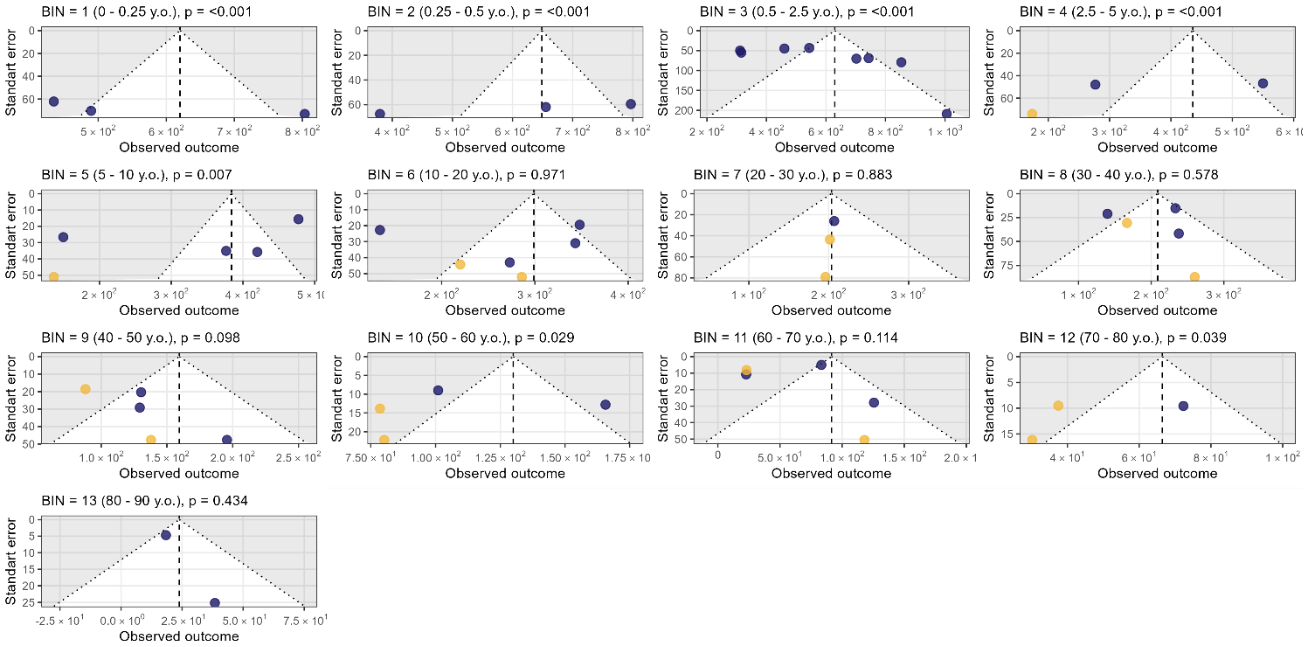

Supplementary Figure 21. Funnel plots for the CD8+ naive T-lymphocyte subpopulation.

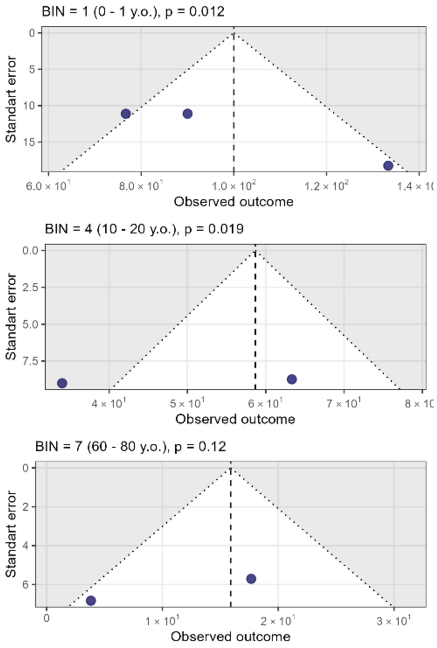

Supplementary Figure 22. Funnel plots for the CD8+ activated T-lymphocyte subpopulation.

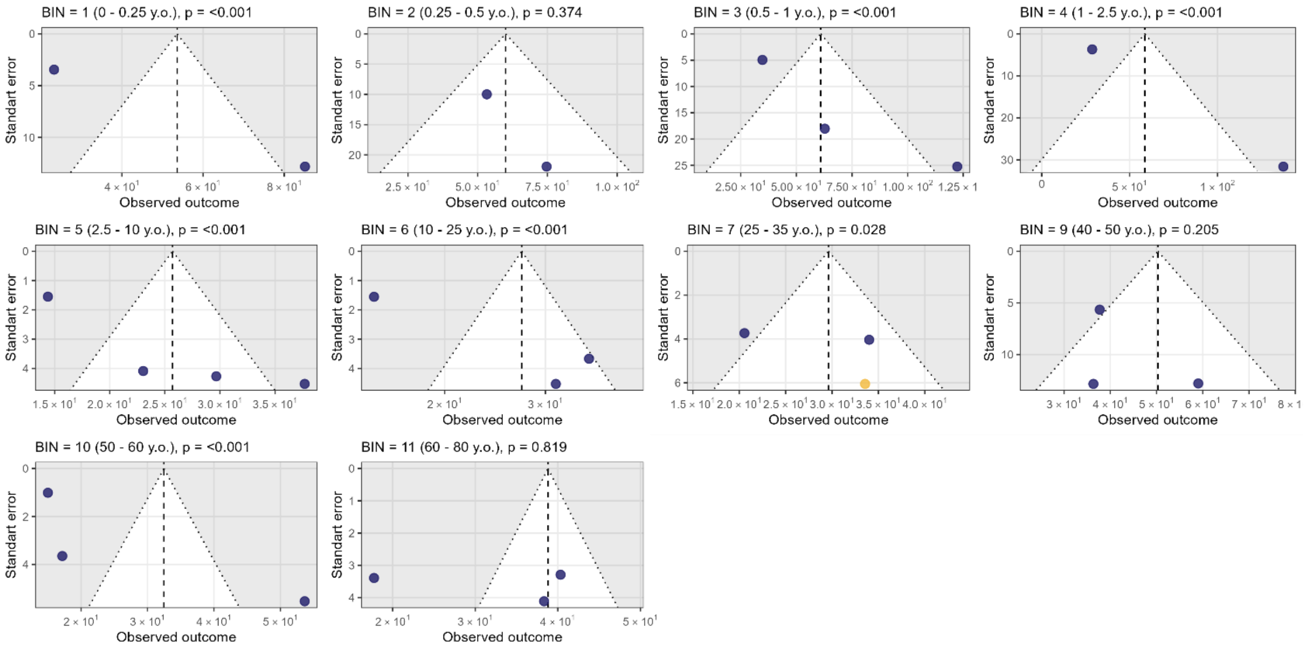

Supplementary Figure 23. Funnel plots for the CD8+ central-memory T-lymphocyte subpopulation.

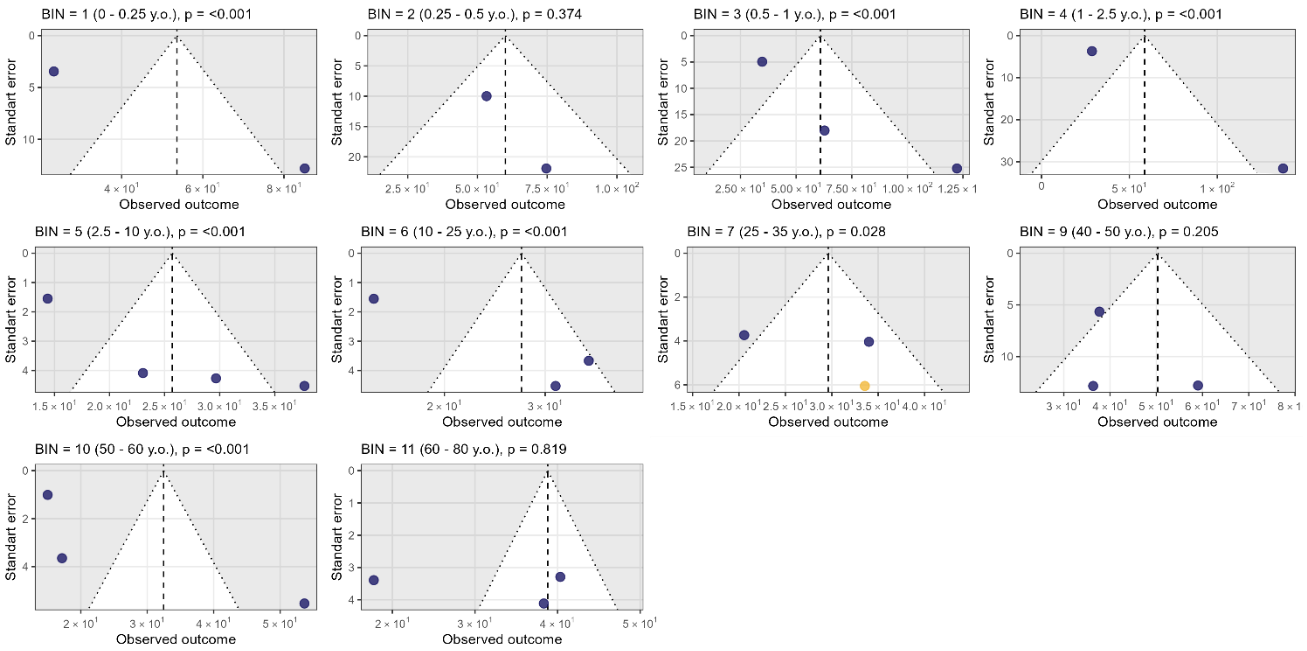

Supplementary Figure 24. Funnel plots for the CD8+ effector-memory T-lymphocyte subpopulation.

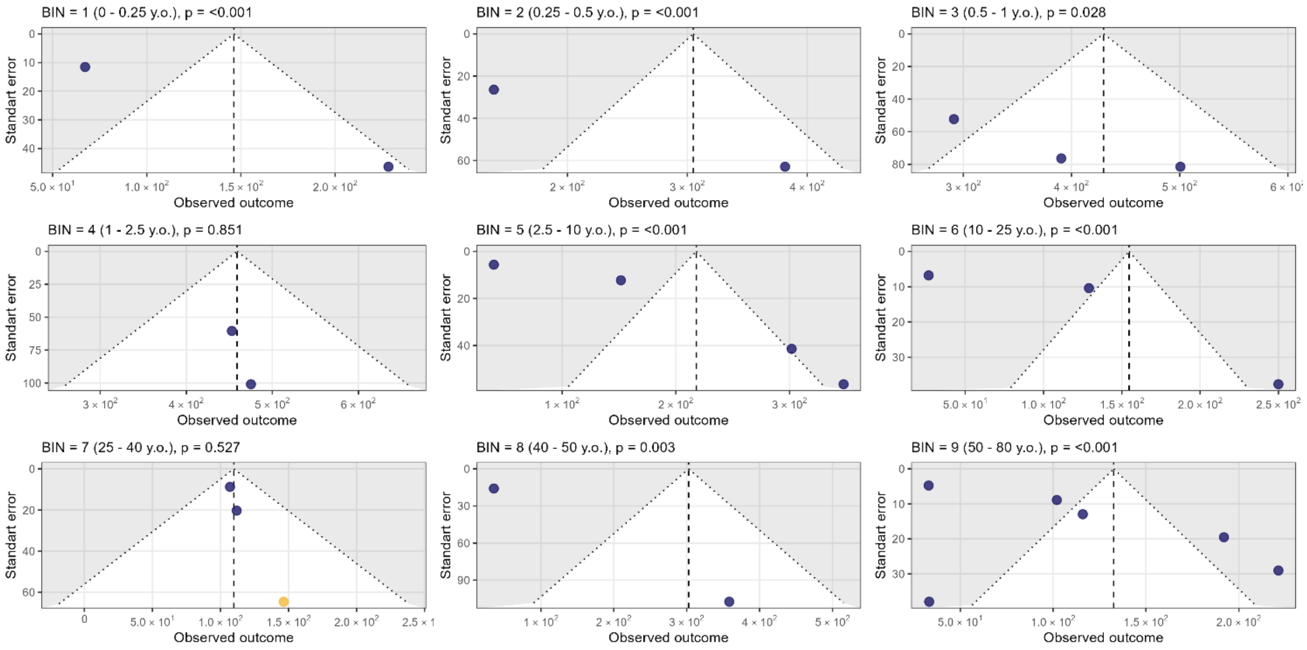

Supplementary Figure 25. Funnel plots for the CD8+ effector T-lymphocyte subpopulation.

For Supplementary Figures 26-28: Dots represent weighted averages calculated using two different weighing methods. Yellow line: identity $y = x$ line.

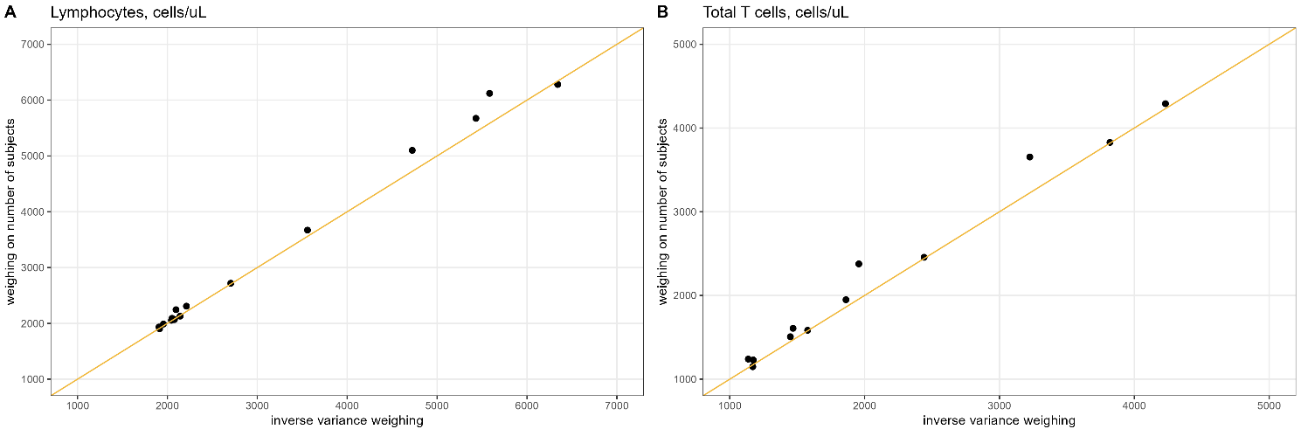

Supplementary Figure 26. Weighing methods; comparative results for total T-lymphocytes subpopulations.

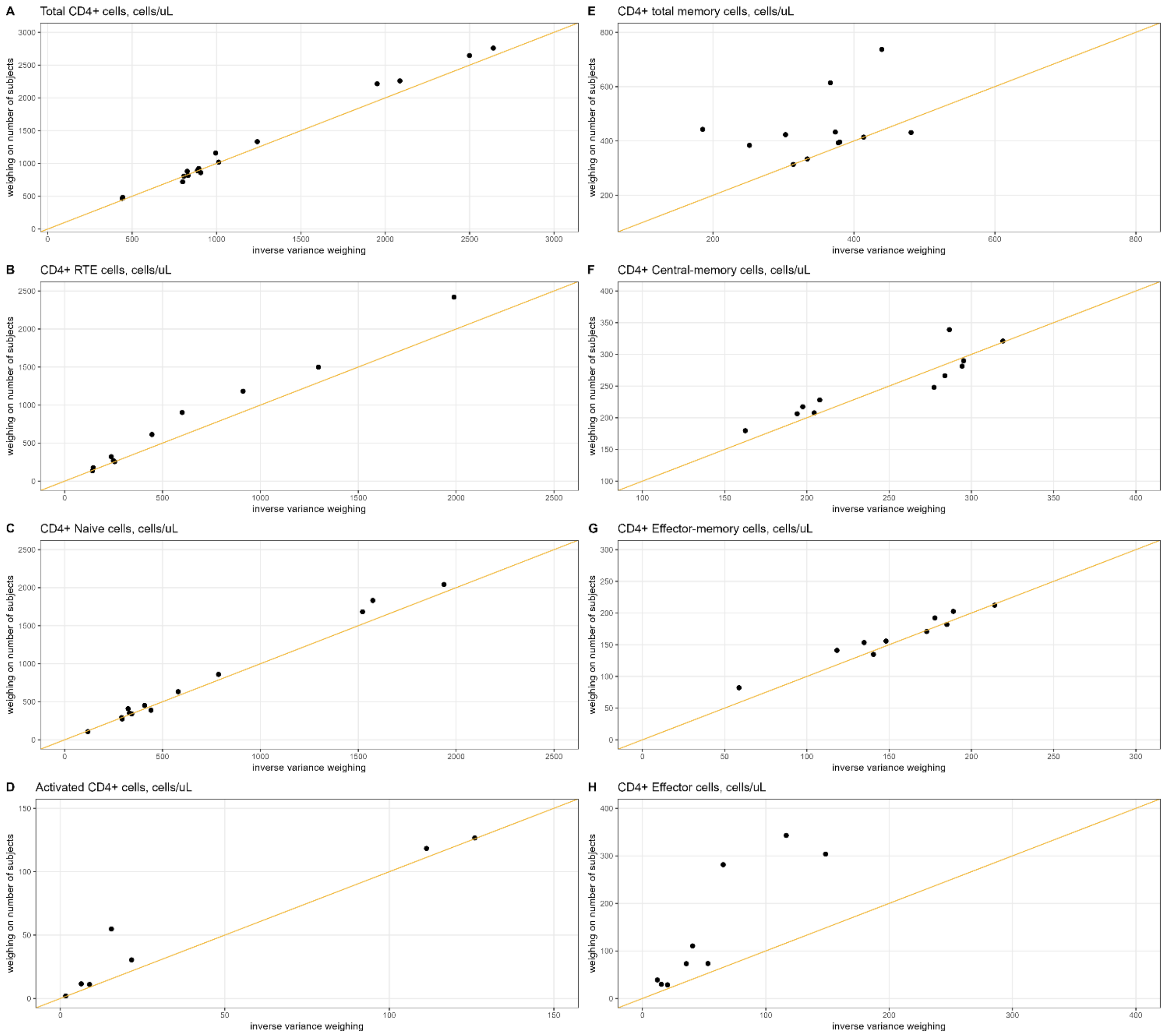

Supplementary Figure 27. Weighing methods; comparative results for CD4+ T-lymphocytes subpopulations.

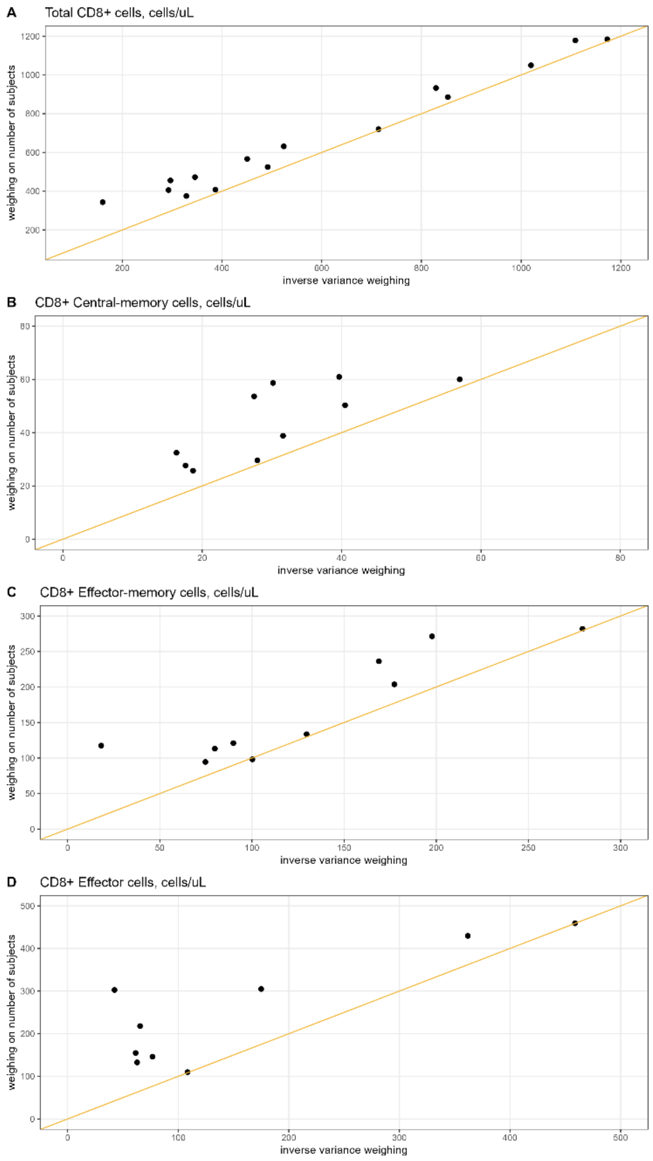

Supplementary Figure 28. Weighing methods; comparative results for CD8+ T-lymphocytes subpopulations.

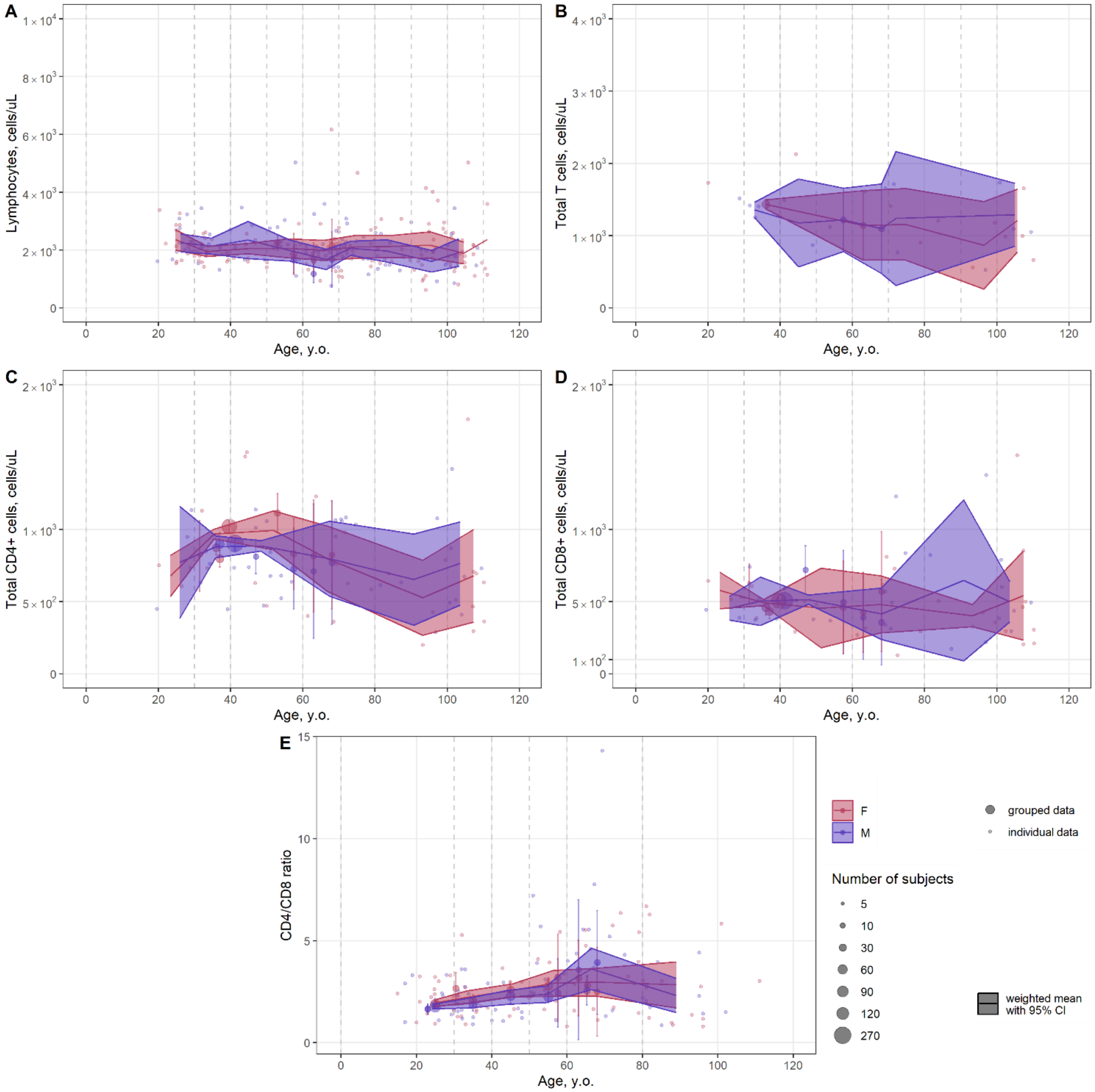

Supplementary Figure 29. Meta-analysis of age-dependent homeostasis of total lymphocyte subpopulations (A – total lymphocytes; B – total CD3+ T-lymphocytes, C – total CD4+ T-lymphocytes, D – total CD8+ T-lymphocytes, E – CD4+/CD8+ ratio), expressed in absolute values, in blood and depending on subjects' sex. Dots represent individual-level data; dots with error bars represent means with 95% CIs of the grouped data; solid lines with shaded areas represent weighted means with 95% CI for each age bin, with bin limits indicated by vertical dashed lines; dot diameters indicate subject numbers in groups; color represents subject sex (F – female, M – male).

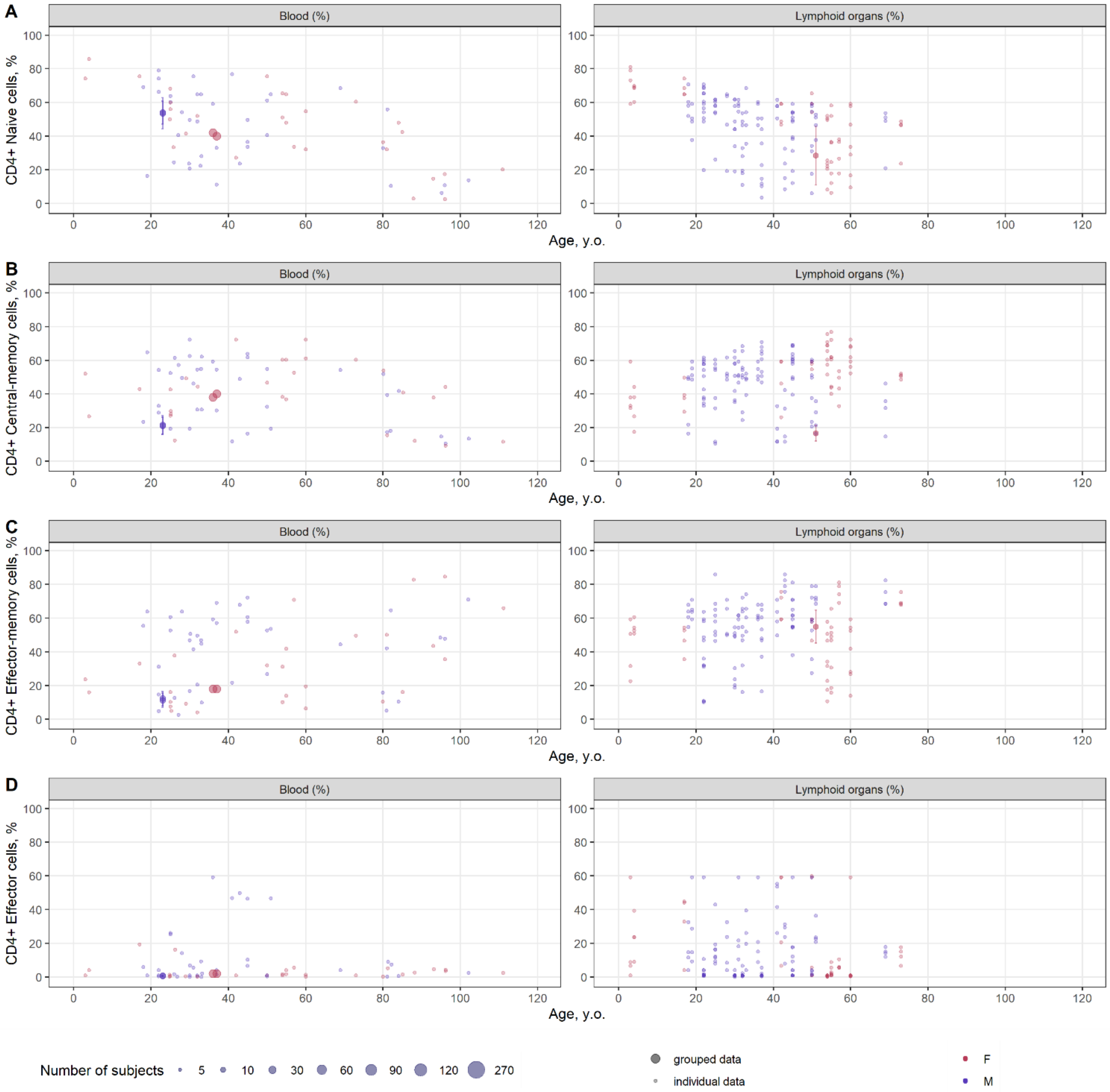

Supplementary Figure 30. Age-dependent homeostasis of CD4+ T-lymphocyte subpopulations in blood and lymphoid organs (values shown are relative to total CD4+ T-lymphocytes) (A – naïve; B – central-memory; C – effector-memory; D – effector) depending on subject's sex. Dots represent individual data, dots with error bars represent means with 95% CIs of the grouped data, dot diameters indicate subject numbers per group; color represents subject sex (F – female, M – male).

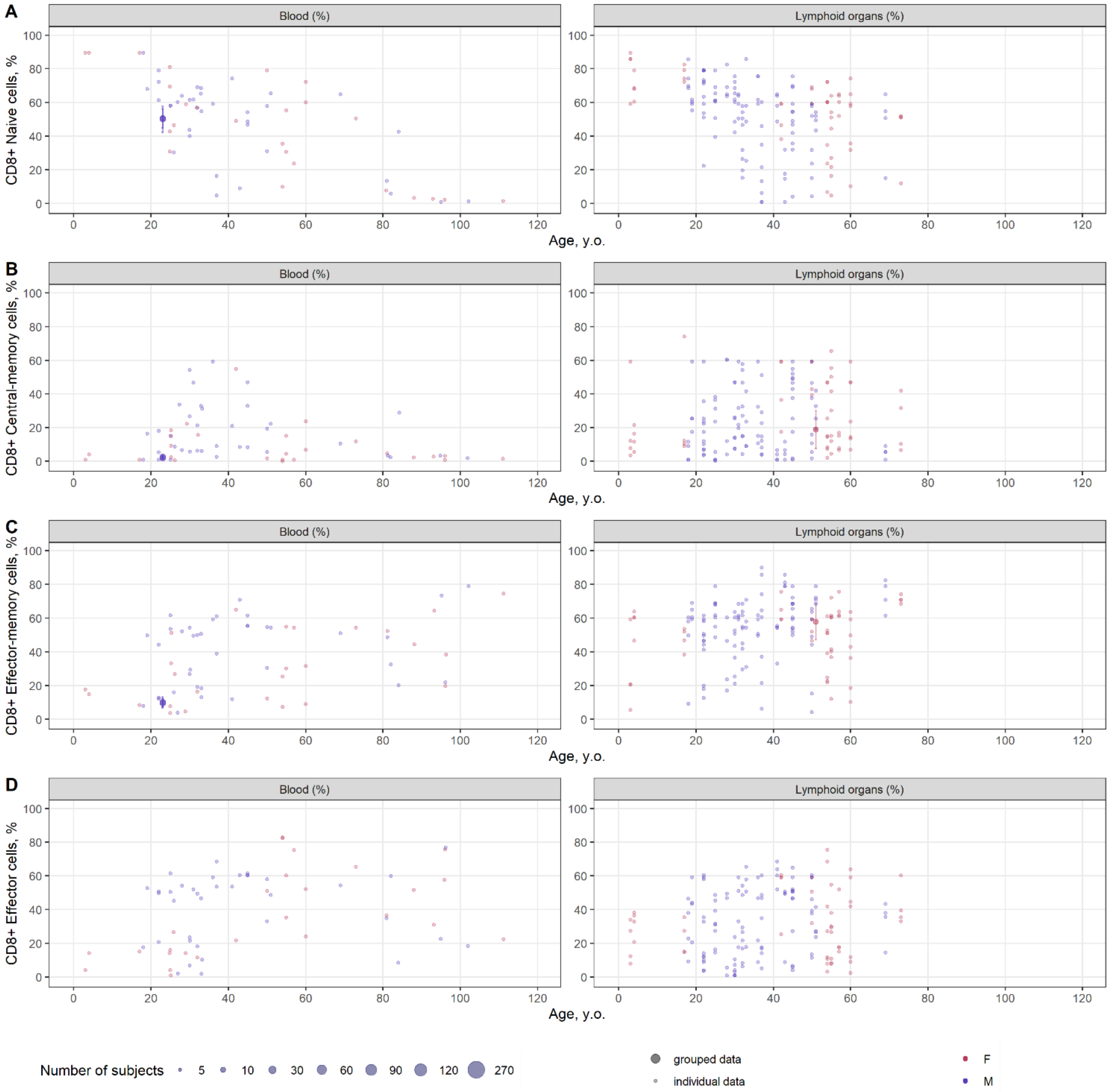

Supplementary Figure 31. Age-dependent homeostasis of CD8+ T-lymphocyte subpopulations in blood and lymphoid organs (values shown are relative to total CD8+ T-lymphocytes) (A – naïve; B – central-memory; C – effector-memory; D – effector) depending on subject's sex. Dots represent individual data, dots with error bars represent means with 95% CIs of the grouped data, dot diameters indicate subject numbers per group; color represents subject sex (F – female, M – male).

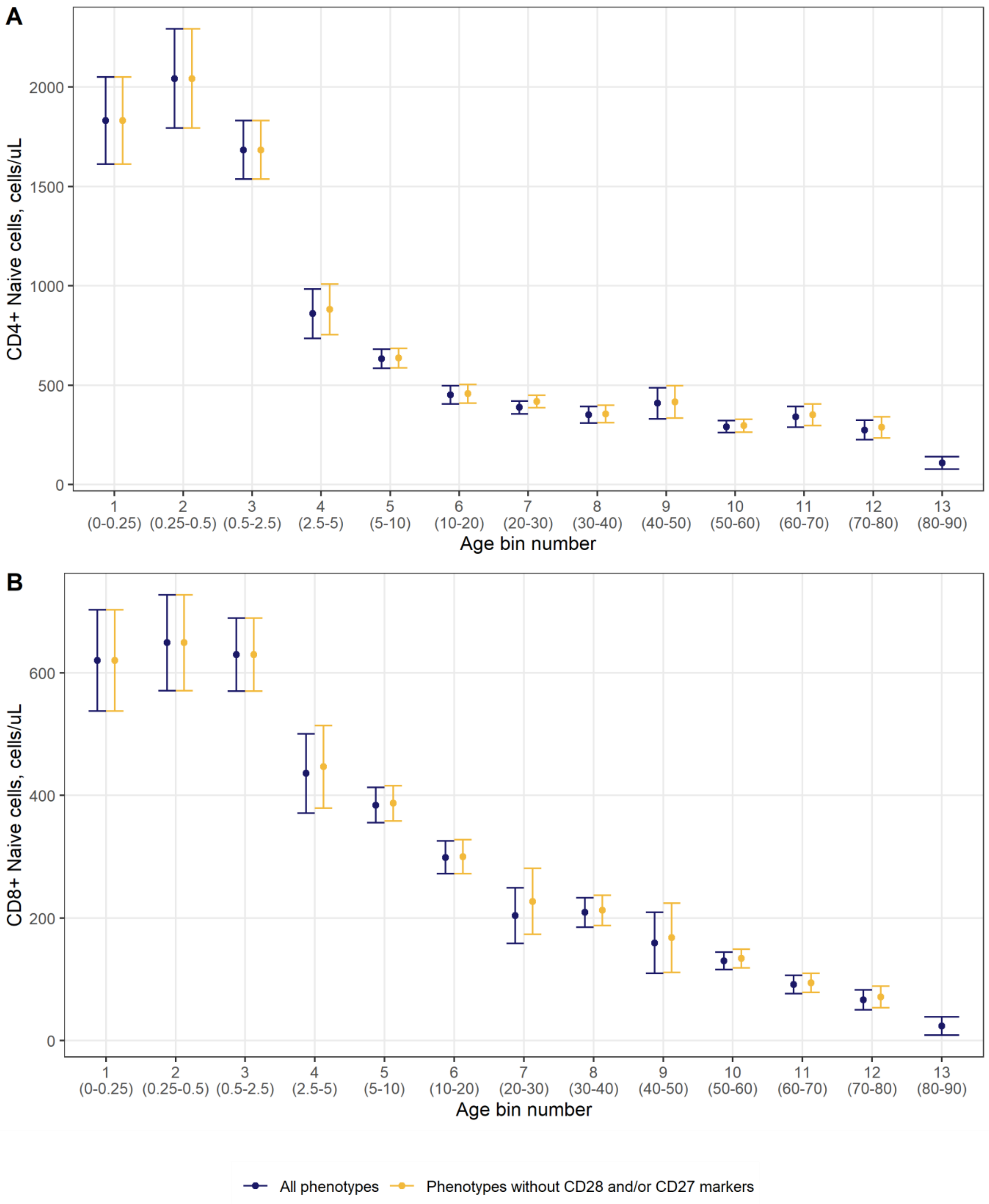

Supplementary Figure 32. Meta-analysis of age-dependent homeostasis of naive subpopulations (A – CD4+; B – CD8+) in blood (expressed in absolute values) depending on the included phenotypes. Dots with error bars represent weighted means with 95% CIs; color represents the used cell

phenotypes (all phenotypes according to Supplementary Table 2 or phenotypes excluding CD27 and CD28 markers); x-axis represents number of age bins with the certain age range in the brackets.

References:

1. Tanaskovic S, Fernandez S, Price P, Lee S, French MA. CD31 (PECAM-1) is a marker of recent thymic emigrants among CD4+ T-cells, but not CD8+ T-cells or γδ T-cells, in HIV patients responding to ART. Immunol Cell Biol. 2010 Mar 1;88(3):321–7.

2. Mahnke YD, Brodie TM, Sallusto F, Roederer M, Lugli E. The who's who of T-cell differentiation: Human memory T-cell subsets. Eur J Immunol. 2013 Nov 1;43(11):2797–809.

3. Cibrián D, Sánchez-Madrid F. CD69: from activation marker to metabolic gatekeeper. Eur J Immunol. 2017 Jun 1;47(6):946–53.
